# Prevalence and Distribution of Axis Subtypes of Refractive Astigmatism in Mexican Outpatients: A Nationwide Multicenter Clinic-Based Cross-Sectional Study

**DOI:** 10.3390/jcm15093522

**Published:** 2026-05-05

**Authors:** José Antonio Magaña-Lizárraga, Abraham García-Gil, Ricardo Daniel Contreras-Espinoza, Eduardo Espinoza-Angulo, Héctor Machado-Jiménez, Marco Antonio Luna-Ruiz-Esparza, Humberto Gómez-Campaña, Abraham Campos-Romero, Jonathan Alcántar-Fernández

**Affiliations:** 1 Innovation and Research Department, Salud Digna, Culiacan 80184, Sinaloa, Mexico; antonio.lizarraga@salud-digna.org (J.A.M.-L.); abraham.gil@salud-digna.org (A.G.-G.); marco.luna@salud-digna.org (M.A.L.-R.-E.); abraham.campos@salud-digna.org (A.C.-R.); 2 Optometry Department, Salud Digna, Culiacan 80184, Sinaloa, Mexico; ricardo.contreras@salud-digna.org (R.D.C.-E.); eduardo.espinoza@salud-digna.org (E.E.-A.); hector.machado@salud-digna.org (H.M.-J.); 3 Medical Direction, Salud Digna, Culiacan 80184, Sinaloa, Mexico; investigacion@salud-digna.org

**Keywords:** refractive error, astigmatism, astigmatism axis orientation, WTR astigmatism, ATR astigmatism, epidemiology, prevalence, children and adolescents, adults

## Abstract

**Background/Objectives**: Astigmatism is one of the most common refractive errors and represents a risk factor for other ocular conditions, including myopia and amblyopia. In Mexico, comprehensive epidemiological data on refractive astigmatism, particularly its axis-related subtypes, remain limited. This study aimed to estimate the prevalence and distribution of axis subtypes of refractive astigmatism in a nationwide, clinic-based cohort of Mexican outpatients. **Methods**: We conducted a retrospective, cross-sectional, multicenter study using anonymized electronic health records (EHRs) from Salud Digna clinics across all 32 states of Mexico. Individuals aged 5–90 years who underwent routine non-cycloplegic eye examinations between 2021 and 2023 were included. Refractive astigmatism was defined as a cylinder power (Cyl) of ≤−0.75 D. Age-adjusted prevalence and distribution of axis subtypes (with-the-rule [WTR], against-the-rule [ATR], and oblique [OBL]) were determined according to sex, age groups, and geographic locations. Logistic regression models evaluated potential associations between demographic variables and refractive astigmatism. **Results**: Data from 8,622,191 individuals were analyzed (median age: 45 years; 63.4% females). The age-standardized prevalence of refractive astigmatism was 41.59% (95% CI: 41.54–41.64%) and was higher in males (45.43%) than females (39.32%). WTR astigmatism predominated among younger individuals, peaking at ages 15–19 (48.97%), whereas ATR astigmatism increased with age, reaching 35.36% among those aged 80–90 years. In multivariable analysis, refractive astigmatism was significantly associated with age, sex, and state of residence, showing a non-linear, non-monotonic age pattern with peaks in adolescence (aOR = 1.47; 95% CI, 1.456–1.484) and older (aOR = 1.177; 95% CI, 1.161–1.193) age, higher odds in males (aOR = 1.269; 95% CI, 1.265–1.272), and marked geographic variability, with the highest in Tlaxcala (aOR = 3.581; 95% CI, 3.491–3.675). **Conclusions**: Refractive astigmatism was more prevalent in males and showed a clear age-related shift in the axis subtype beginning in the early 40s. These findings provide large-scale, real-world evidence that enhances the understanding of astigmatism epidemiology in Mexico and may inform future research and public health planning.

## 1. Introduction

Globally, refractive errors are the primary factors leading to visual impairment and blindness [1]. Astigmatism is one of the most common and complex forms of refractive error, contributing significantly to the global burden of visual impairment. This condition is characterized by a variation in refractive power across different ocular meridians, resulting in the creation of two perpendicular focal lines rather than a single focal point on the retina. This phenomenon is primarily attributable to the asymmetrical curvature of the cornea or crystalline lens, which results in blurred or distorted vision at both near and far distances, thereby exerting a substantial influence on individuals’ visual-related and overall quality of life [2,3,4].

In general, astigmatism is classified as regular or irregular [4]. Based on the orientation of the corneal meridian axis, regular astigmatism is further subclassified into with-the-rule (WTR), against-the-rule (ATR), and oblique (OBL) astigmatism [4]. Recent findings have demonstrated an association between specific astigmatism axis subtypes and axial elongation of children’s eyes, suggesting that early astigmatic axis patterns may influence ocular growth and impair refractive development [5]. Furthermore, its association with the development of amblyopia, strabismus, and myopia, coupled with its increased prevalence in the pediatric population, has positioned astigmatism as both a clinical challenge and a public health concern [6,7,8].

Globally, it is estimated that astigmatism affects 14.9% of children and 40.4% of the adult population [9]. It is well documented that a range of demographic and geographical factors, including age, sex, ethnicity, and regional or national location, influence the prevalence and distribution of this condition [10,11,12,13,14,15,16]. Likewise, several studies have described variations in the astigmatism axis, underscoring the differences in its prevalence, distribution, and dynamic characteristics across children and adult populations [17,18,19,20,21]. Although previous studies have documented the overall prevalence of refractive astigmatism in Mexico, covering both children and adult populations at the local [22,23,24,25,26], regional [27], and national [28] levels, specific aspects related to astigmatism axis orientation, such as its prevalence, age-related changes, and geographic distribution, remain largely unexplored.

To address this research gap, we conducted a large-scale, multicenter study aimed at estimating the prevalence and distribution of axis subtypes of refractive astigmatism in a nationwide, clinic-based cohort of Mexican outpatients and describing its epidemiological patterns according to sex, age, and state of residence. These findings provide robust real-world evidence from a large nationwide outpatient cohort that enhances the current epidemiological knowledge of astigmatism in Mexico and may support future clinical and epidemiological research and inform public health planning aimed at addressing the burden of astigmatism in the country.

## 2. Materials and Methods

Salud Digna is a private, not-for-profit, and non-governmental primary care institution that offers diagnostic services to the general population in Mexico, regardless of their socioeconomic status or social security coverage. The present research followed the Strengthening the Reporting of Observational Studies in Epidemiology (STROBE) reporting guideline for cross-sectional studies [29].

### 2.1. Study Design and Population

This retrospective, nationwide, multicenter, clinic-based, cross-sectional study analyzed the electronic health records (EHRs) of individuals aged 5–90 years who underwent routine eye examinations at Salud Digna outpatient clinics across all 32 states of Mexico between 2 January 2021, and 31 December 2023. Salud Digna provides comprehensive and standardized routine eye examinations conducted by certified optometrists at its clinics across Mexico.

Briefly, patient identity was verified, and clinical history was recorded utilizing a proprietary electronic clinical form as part of standard practice. Interpupillary and nasopupillary distances were measured, and uncorrected visual acuity was assessed using digital optotypes at a 3 m distance (CS Pola 600, CS 550, Essilor Instruments, Paris, France). Non-cycloplegic autorefraction (AKR550 and WAM 800; Essilor Instruments, Paris, France), followed by subjective refinement, was performed to determine the final refractive prescription and to evaluate both distance and near visual acuity. Examination findings and recommendations were communicated to the patients, and referrals for ophthalmology or retinal imaging were provided when clinically indicated.

In this study, participants were selected through a convenience sampling method. Anonymized EHRs were queried to extract demographic data (sex, age, and state of residence), ocular clinical history, and refraction test data (spherical power, cylindrical power, and axis) from both eyes. A total of 9,862,505 records were initially retrieved. To ensure data consistency and prevent duplication, only the most recent examination was included in the analysis for individuals with multiple assessments. Individuals with a history of ocular surgery, ocular trauma, or self-reported ocular pathologies, including cataracts, glaucoma, diabetic or hypertensive retinopathy, and age-related macular degeneration, were also excluded based on information routinely recorded during ocular examinations at all clinics. Finally, records with incomplete data on any of the variables required for analysis (age, sex, state of residence, or refraction eye measurement) were excluded from the final dataset using a complete-case analysis approach.

Because of the strong inter-eye correlation in spherical and cylindrical refraction across the total dataset (Spearman’s correlation coefficients: *ρ* = 0.95 and *ρ* = 0.83, respectively; both *p* < 0.001), only right-eye data were considered for further analyses. For analytical purposes, individuals were stratified into the following age groups: 5–9, 10–14,15–19, 20–29, 30–39, 40–49, 50–59, 60–69, 70–79, 80–90 years.

### 2.2. Definitions of Astigmatism

Cylindrical refractive error was recorded in the minus cylinder format. To facilitate comparison with other studies [9,30,31] and to capture clinically relevant astigmatism [32], refractive astigmatism was defined as a cylinder power (Cyl) ≤ −0.75 diopters (D) at any axis. Furthermore, astigmatism was classified into three subtypes according to the cylindrical axis orientation: with-the-rule (WTR; 180° ± 30° or 0° ± 30°), against-the-rule (ATR; 90° ± 30°), and oblique (OBL; 31–59° and 121–149°) [2,33,34]. The spherical equivalent (SE) was calculated as the sphere power plus one-half of the cylinder power.

### 2.3. Ethics Statement, Consent, and Permissions

The Research and Ethics Committee of Salud Digna (SDI-202502) granted approval for this study, which was conducted in strict accordance with the guidelines for clinical management information, the Declaration of Helsinki, and the relevant national regulations of Mexico, including the Federal Law on Data Privacy Protection. Consent for the utilization of health record information was secured in compliance with the Mexican Federal Law on Personal Data Protection Held by Private Parties (LFPDPPP, by its Spanish acronym; https://www.diputados.gob.mx/LeyesBiblio/pdf/LFPDPPP.pdf; accessed on 12 January 2026).

Individuals who utilize the services of Salud Digna clinics consent to the institution’s privacy policy, which encompasses the utilization of anonymized data for research purposes. Consequently, the Research and Ethics Committee of Salud Digna has waived the requirement for individual informed consent for this retrospective analysis of electronic health records, in accordance with the Mexican Federal Law on the Protection of Personal Data Held by Private Parties. To ensure data protection, unique identification codes were assigned, which served to protect individual privacy and eliminate the possibility of duplication in future analyses.

### 2.4. Statistical Analysis

All statistical analyses were performed using the open-source R Statistical Software (v.4.4.2; R Core Team 2025) [35]. The Anderson–Darling test was used to evaluate the normality of all continuous data in the study population. Since the data did not follow normal distribution, non-parametric statistical tests were utilized. For statistical description, categorical variables were presented as counts and percentages, whereas continuous variables were described using medians along with interquartile ranges (IQRs). Refraction error data were compared between the sexes using the Mann–Whitney U test, with the rank-biserial correlation coefficient (*r*) used to estimate the effect size.

Prevalence estimates of refractive astigmatism and its axis-associated subtypes were reported as percentages with 95% confidence intervals (CIs) calculated using the Clopper–Pearson exact method. The Chi-square test of independence was used to assess the associations between sex and the presence of refractive astigmatism, as well as its axis-associated subtypes. Effect sizes were calculated using Cramér’s *V* coefficient. Additionally, overall and state-level prevalence estimates were age-adjusted using the direct standardization method and the World Health Organization (WHO) standard world population [36]. The Z-test was employed to analyze the differences in age-adjusted estimates related to sex.

Univariable and multivariable logistic regression analyses were performed to evaluate the association between refractive astigmatism and demographic variables, including age, sex, and state of residence, as covariates. These variables were included based on their availability in the EHRs and a priori epidemiological relevance. Given the observed non-linear relationship between age and refractive astigmatism, age was modeled as a categorical variable using predefined age groups, with the 5–9 years group as the reference category. Female sex and Chiapas, the state with the lowest observed prevalence, were specified as reference categories.

A forward stepwise strategy was employed for model selection, with the Akaike Information Criterion (AIC) serving as the guiding metric, with the model including age, sex, and state of residence providing the best fit; therefore, it was selected as the final model. The state of residence was included as a fixed-effect covariate to account for geographic heterogeneity, and no additional clustering adjustment was performed. Adjusted odds ratios (aORs) along with their 95% confidence intervals (CIs) were calculated using the multivariable model. A two-sided *p*-value < 0.05 was considered to indicate statistical significance across all analyses.

## 3. Results

### 3.1. Population Characteristics

After data cleaning, a final dataset of 8,622,191 unique records from the right-eye data was retained for retrospective analysis. Table 1 summarizes the distribution and refraction error data observed in the Mexican outpatient cohort. The median age of the study population was 45 years (IQR, 25–57). The proportion of females and males was 63.4% and 36.6%, respectively, resulting in a female-to-male ratio of 1.73:1. The median age of females was 45 years (IQR, 26–57), whereas for males, it was 44 years (IQR, 24–57). Regarding refractive error data, the spherical equivalent (SE) ranged from −25.00 to +18.00 D, with a median of 0.00 D (IQR, −1.00–+1.00). Cylinder power (Cyl) ranged from −6.50 to 0.00 D with a median of −0.50 D (IQR, −1.00–0.00).

The SE and Cyl values were comparable between the sexes (Table 1). The median SE was 0.00 D (IQR, −0.88–+1.12) in females and −0.12 D (IQR, −1.00–+0.75) in males, while the median Cyl was −0.50 D (IQR, −1.00–0.00) in females and −0.50 D (IQR, −1.25–0.00) in males. The Mann–Whitney U test revealed statistically significant differences in the dispersion of both SE and Cyl between the sexes (*p* < 0.001); however, the effect sizes were small in magnitude (*r* < 0.1), suggesting that these differences are unlikely to be clinically relevant.

### 3.2. Prevalence of Refractive Astigmatism

A total of 3,361,040 individuals were identified as astigmatic individuals, of whom 60.72% and 21.19% concomitantly manifested myopic (SE ≤ −0.50 D) or hyperopic (SE ≥ +0.50 D) spherical equivalent refractive error, respectively. Moreover, astigmatism was predominantly composed of the WTR (67.28%), followed by the ATR (26.21%) and OBL (6.51%) subtypes (Table 1).

Overall, the age-standardized prevalence of refractive astigmatism was 41.59% (95% CI, 41.54–41.64%), with a higher prevalence rate in males (45.35%; 95% CI, 45.27–45.43%) compared to females (39.32%; 95% CI, 39.25–39.38%). This sex difference was statistically significant (Z = 114.6, *p* < 0.001). Regarding astigmatism axis orientation, the age-standardized prevalence rates for WTR, ATR, and OBL astigmatism were 32.04% (95% CI, 31.99–32.09%), 7.34% (95% CI, 7.33–7.36%), and 2.20% (95% CI, 2.19–2.21%), respectively.

The age-adjusted prevalence of astigmatism axis subtypes differed significantly between the sexes. Among females, the estimated prevalence of WTR, ATR, and OBL astigmatism was 30.45% (95% CI, 30.39–30.51%), 6.75% (95% CI, 6.73–6.77%), and 2.12% (95% CI, 2.11–2.13%), respectively. In males, the corresponding prevalences were 34.59% (95% CI, 34.51–34.67%), 8.41% (95% CI, 8.38–8.44%), and 2.35% (95% CI, 2.33–2.37%). These differences were statistically significant for all subtypes (WTR Z = 81.14; ATR Z = 90.24; OBL Z = 20.16; all *p* < 0.001).

### 3.3. Prevalence of Refractive Astigmatism Across Different Age Groups

The overall prevalence of refractive astigmatism in children and adolescents aged <20 years was 49.15% (95% CI, 49.06–49.23%). In contrast, among adults aged ≥20 years, the prevalence was 37.01% (95% CI, 36.98–37.05%). Table 2 shows the crude prevalence estimates of refractive astigmatism stratified by age and sex within the Mexican outpatient cohort. Overall, prevalence estimates steadily increased with age, peaking at 15–19 years (51.49%; 95% CI, 51.37–51.61%).

**Figure 1:**
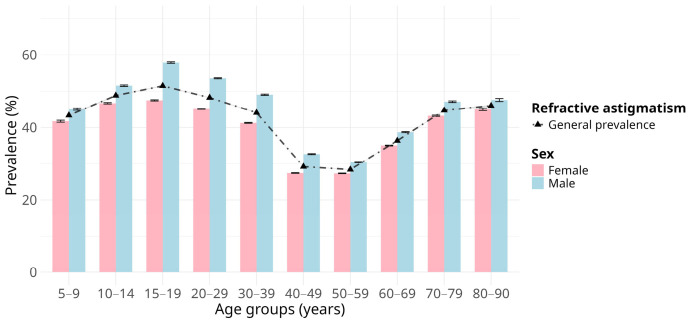
Age group sex-specific prevalence of refractive astigmatism among Mexican outpatients. Bar chart representing the crude prevalence estimates of refractive astigmatism in female and male individuals across age groups. Error bars indicate the 95% confidence interval for the prevalence estimates. The dot-dashed line corresponds to the trend of the overall prevalence estimate of refractive astigmatism across age groups.

The age groups most affected by astigmatism were 10–14, 15–19, and 20–29 years. Figure 1 depicts the age- and sex-specific trends in the prevalence of refractive astigmatism. As age increased, a noticeable decreasing trend was observed, stabilizing among individuals aged 40–59 years (29.12–28.32%), followed by an increase in those aged 60 and older (36.33–46.02%). The prevalence of refractive astigmatism varied significantly between the sexes across all age groups, being predominantly higher in males (*p* < 0.001) (Table 2). However, the corresponding effect sizes were uniformly small (φ_c_ < 0.1) across all comparisons.

**Figure 2:**
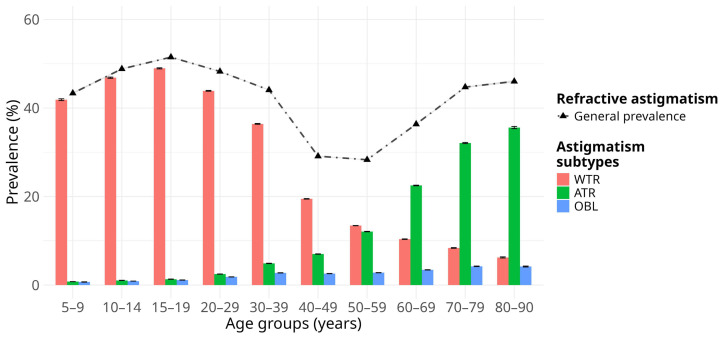
Age group-specific prevalence of astigmatism subtypes regarding the meridional axis among Mexican outpatients. Bar chart represents the crude prevalence estimates of astigmatism axis subtypes across the age groups. Error bars indicate the 95% confidence interval for the prevalence estimates. The dot-dashed line corresponds to the trend of the overall prevalence estimate of refractive astigmatism across age groups. WTR, with-the-rule astigmatism; ATR, against-the-rule astigmatism; OBL, oblique astigmatism.

As shown in Figure 2, the astigmatism axis subtypes varied significantly with age. WTR astigmatism followed a steady increase in prevalence among individuals younger than 30 years, peaking at 15–19 years (48.97%; 95% CI, 48.84–49.09%), followed by a clear descending trend after the age of 30 years and older (36.39–6.25%). Conversely, a reversed trend was observed for ATR astigmatism with advancing age, reaching a higher prevalence in individuals aged 60 years and older (22.52–35.56%). Notable changes in the trends of WTR and ATR astigmatism occurred between the ages of 50 and 59 years. OBL astigmatism exhibited a slight but consistent increase with age, mainly affecting older adults (Figure 2 and Table S1).

Overall, the prevalence of each astigmatism axis subtype differed significantly between the sexes across all age groups, with males exhibiting the highest prevalence rates (Table S2). However, this pattern was reversed in individuals aged 60 years and older, among whom females showed a slightly, yet statistically significant, higher prevalence, particularly for WTR and OBL astigmatism. The corresponding effect sizes were uniformly small (φ_c_ < 0.1) for all comparisons.

### 3.4. Geographical Distribution of Refractive Astigmatism

**Figure 3:**
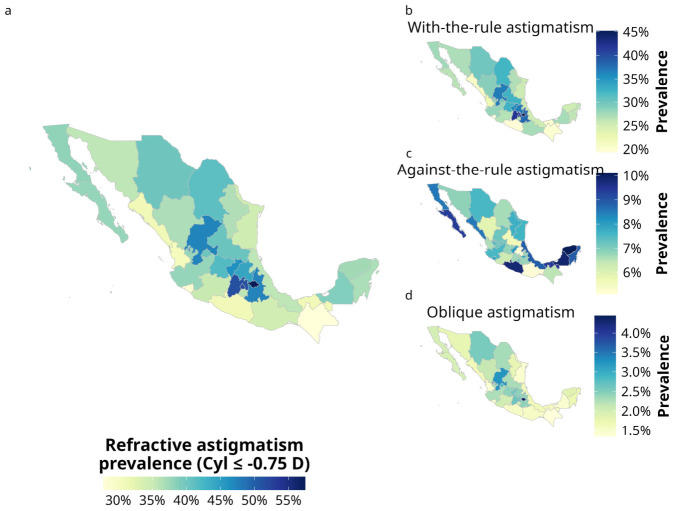
Geographic distribution of age-adjusted prevalence of refractive astigmatism in the Mexican outpatient cohort. Panel (**a**) illustrates the state-level prevalence of refractive astigmatism. Panels (**b**–**d**) display the spatial distribution of specific astigmatism subtypes categorized by cylindrical axis orientation: with-the-rule, against-the-rule, and oblique astigmatism, respectively.

Figure 3 depicts the geographic distribution of refractive astigmatism and its axis-associated subtypes across Mexico. The age-adjusted prevalence of refractive astigmatism ranged from 27.84% to 57.42% across all 32 states. The highest prevalence rates were noted in Tlaxcala (57.42%; 95% CI, 56.44–58.41%), Mexico City (52.04%; 95% CI, 51.87–52.21%), and the State of Mexico (51.37%; 95% CI, 51.23–51.51%), whereas the lowest rates were in Chiapas (27.84%; 95% CI, 27.55–28.13%), Colima (28.71%; 95% CI, 28.22–29.2%), and Nayarit (30.55%; 95% CI, 30.13–30.97%) (Figure 3a and Table S3). When analyzed by sex, the rates were more prevalent among males across all states (*p* < 0.001); however, the effect sizes were small in magnitude (φ_c_ < 0.1) (Table S3).

The geographic distribution of astigmatism axis subtypes showed slight variation across states compared to the overall age-adjusted national distribution of refractive astigmatism. WTR astigmatism was most prevalent in Tlaxcala (45.16%; 95% CI, 44.26–46.07%), Mexico City (42.6%; 95% CI, 42.44–42.76%), and the State of Mexico (42%; 95% CI, 41.88–42.13%). In contrast, it was least prevalent in Colima (19.36%; 95% CI, 18.92–19.8%), Guerrero (19.63%; 95% CI, 19.2–20.08%), and Chiapas (20.05%; 95% CI, 19.79–20.31%) (Figure 3b). WTR astigmatism was more prevalent among males across all states; however, in seven of the 32 states, the sex-specific differences did not reach statistical significance (*p* > 0.05) (Table S4).

The highest prevalence rates for ATR astigmatism were observed in Yucatan (10.01%; 95% CI, 9.93–10.28%), Campeche (9.87%; 95% CI, 9.54–10.22%), and Guerrero (9.8%; 95% CI, 9.6–10.01%), whereas the lowest were found in Hidalgo (5.1%; 95% CI, 4.94–5.27%), Oaxaca (5.15%; 95% CI, 5.0–5.32%), and San Luis Potosi (5.6%; 95% CI, 5.48–5.72%) (Figure 3c). When analyzed by sex, ATR astigmatism was more prevalent among males across all states; however, in six of the 32 states, the differences observed between sexes did not reach statistical significance (*p* > 0.05) (Table S5). Prevalence rates exceeding 8.5% were concentrated in states bordering the Gulf regions. Excluding Tamaulipas, the prevalence in the Gulf of Mexico states ranged from 8.87% to 10.01%, whereas among the Gulf of California states, excluding Sonora, it ranged from 8.51% to 9.34% (Figure 3c and Table S5).

The prevalence rates of OBL astigmatism were below 2.5% in most states. In Tlaxcala, the prevalence was highest at 4.44% (95% CI, 4.19–4.71%). This was followed by Zacatecas, Mexico City, Aguascalientes, the State of Mexico, Queretaro, and Chihuahua, where rates ranged narrowly from 2.56% to 3.23% (Figure 3d). Although OBL astigmatism was predominantly higher among males, no statistically significant sex differences were observed in eight of the 32 states (*p* > 0.05) (Table S6). Overall, effect sizes were consistently small (φ_c_ < 0.1) across sex-specific comparisons for the WTR, ATR, and OBL astigmatism subtypes.

### 3.5. Factors Associated with Refractive Astigmatism

Table 3 shows the results of the univariable and multivariable logistic regression analyses. Univariable analysis revealed significant associations between age, sex, or state of residence and refractive astigmatism (all *p* < 0.001). These associations remained statistically significant after multivariable adjustment. A non-linear and non-monotonic relationship between age and refractive astigmatism was confirmed. Compared with children aged 5–9 years, the odds of refractive astigmatism were significantly higher in adolescents and young adults, peaking at 15–19 years age group (aOR = 1.47; 95% CI, 1.456–1.484; *p* < 0.001), followed by those aged 20–29 years (aOR = 1.299; 95% CI, 1.288–1.311; *p* < 0.001) and 10–14 years (aOR = 1.283; 95% CI, 1.27–1.295; *p* < 0.001). A modest increase persisted in individuals aged 30–39 years (aOR = 1.088; 95% CI, 1.078–1.098; *p* < 0.001).

In contrast, significantly lower odds were found among individuals aged 40–49 years (aOR = 0.580; 95% CI, 0.575–0.585; *p* < 0.001) and 50–59 years (aOR = 0.551; 95% CI, 0.546–0.556; *p* < 0.001). This pattern attenuated in individuals aged 60–69 years (aOR = 0.796; 95% CI, 0.789–0.803; *p* < 0.001) and reversed in older age groups, with increased odds in those aged 70–79 years (aOR = 1.119; 95% CI, 1.108–1.13; *p* < 0.001) and 80–90 years (aOR = 1.177; 95% CI, 1.161–1.193; *p* < 0.001). Male sex was independently associated with higher odds of refractive astigmatism compared with female sex (aOR = 1.269; 95% CI, 1.265–1.272; *p* < 0.001).

Regarding state of residence, a marked geographic variation was observed, mirroring the prevalence patterns of refractive astigmatism. Using Chiapas as the reference category, all states showed significantly higher odds of refractive astigmatism (all *p* < 0.001). The highest odds were observed in Tlaxcala (aOR = 3.581; 95% CI, 3.491–3.675), Mexico City (aOR = 2.77; 95% CI, 2.74–2.801), and the State of Mexico (aOR = 2.72; 95% CI, 2.69–2.749). Other states with notably elevated odds included Puebla (aOR = 2.356; 95% CI, 2.327–2.385), Zacatecas (aOR = 2.362; 95% CI, 2.316–2.407), Queretaro (aOR = 2.319; 95% CI, 2.289–2.35), and Hidalgo (aOR = 2.013; 95% CI, 1.974–2.052). Moderate increases were observed across the remaining states, with aORs ranging from 1.136 to 1.885.

## 4. Discussion

Astigmatism is the most reported refractive error worldwide, with a wide distribution and considerable variability in prevalence, depending on the study population and geographic region [9,30,31]. The visual and functional impacts of astigmatism are extensively documented in the literature and have been summarized by Read et al. [2]. Clinically, astigmatism has been linked with diverse ocular and visual disorders throughout the lifespan. During childhood development, astigmatism is frequently associated with meridional amblyopia, strabismus, and myopia, all of which may hinder visual maturation and academic performance if left uncorrected [2,5,6,7,8].

In adulthood, uncorrected astigmatism can significantly impair functional vision, contributing to a reduced performance in low-light environments (night-time driving), an increased risk of falls, and diminished efficiency in visually demanding occupational tasks, such as reading, digital editing, or screen-based work. These visual limitations not only affect the quality of life but may also contribute to occupational productivity losses among working-age individuals and, in older adults, may increase the risk of injury-related morbidity and mortality, particularly due to falls [2,31,37]. Epidemiological data on astigmatism in Mexico are limited, and existing studies report significant variability in prevalence rates, primarily attributable to inconsistencies in factors such as age, sex, geographic region, and methodological constraints [38].

While several international studies have examined diverse epidemiological aspects of astigmatism, such as magnitude, severity, geographical distribution, and classification based on spherical refraction or axis orientation, comparable investigations in Mexico remain limited. Currently, no studies have comprehensively analyzed epidemiological aspects beyond the overall prevalence and geographic distribution within the Mexican population. Only three studies have partially addressed some of these aspects: one focused on broad age-range prevalence at the regional level [27], while two others examined astigmatism prevalence in relation to spherical refractive error in general and school-aged populations at the regional and national levels [23,28].

As no studies to date in Mexico have specifically focused on astigmatism axis orientation, the prevalence and distribution of with-the-rule (WTR), against-the-rule (ATR), and oblique (OBL) astigmatism remain unclear. In this study, we investigated the prevalence of refractive astigmatism and its axis-associated subtypes using data from 8.62 million Mexican outpatients across a broad age spectrum, collected between 2021 and 2023. To the best of our knowledge, this research represents the first large-scale, nationwide, multicenter initiative to offer a comprehensive and up-to-date overview of the prevalence and distribution of refractive astigmatism and its axis-associated subtypes in the Mexican population.

Because astigmatism prevalence varies significantly with age and the studied dataset exhibited a bimodal age distribution, we used direct age standardization with the WHO world standard population to correct for potential age-related bias in the prevalence estimates. However, this methodological approach limits the comparability of our age-adjusted rates with those from previous studies that employed different standard populations or did not adjust for age, thereby precluding direct comparisons between studies.

Despite this, refractive astigmatism, defined as a cylindrical power threshold of ≤−0.75 D, was predominantly observed in 38.98% of the Mexican outpatient cohort, with an age-adjusted prevalence rate of 41.59% (95% CI, 41.54–41.64%). This finding aligns with previous international research [11,14,15,16,39,40,41] and studies conducted in Mexico [22,23,26,28], all of which have consistently identified astigmatism as the predominant refractive error among both children and adult populations. Likewise, the overall prevalence rate observed here is consistent with findings from other populations in Asia, such as Singapore [10,42,43], China [15,44], and Iran [19], and the Americas, including the United States [11] and Panama [45], although some variability in prevalence rates exists across different demographic and methodological contexts.

In contrast to findings from various studies conducted in Mexico, our estimated prevalence rate significantly exceeds those reported by Gomez-Salazar et al. [27] for 6- to 90-year-old outpatients (13.5%), Signes-Soler et al. [22] for 5- to 14-year-old schoolchildren from Quintana Roo (22.3%), and Teran et al. [24,25,26] for elementary-, middle-, and high-school children from Sinaloa (18.63–29.17%). However, it remains lower than the estimates provided by Ramírez-Ortiz et al. [28] for 6- to 12-year-old schoolchildren (54.6%) and by Barba-Gallardo et al. [23] for child/adolescent (61.1%) and adult (57.1%) populations.

These discrepancies in reported prevalence rates may be attributed to several factors, including demographic, geographical, and sample size differences among the studied population groups, as well as variations in ocular examination techniques and sampling methodologies. For example, we calculated age-adjusted prevalence estimates to address age-related biases, a method not utilized in previous studies. The absence of such standardization in earlier research could introduce biases due to the differing age structures of the populations examined. Moreover, variations in the cylindrical power thresholds used to define astigmatism are well documented to considerably influence and affect the reported prevalence rates of this condition [19,21,38,44,46]. Consequently, direct comparisons are not warranted, and the results should be interpreted with appropriate caution.

Our study revealed sex-specific differences and age-related patterns in the prevalence of refractive astigmatism and axis-associated subtypes of astigmatism. Irrespective of age group or state-level stratification, males consistently exhibited significantly higher rates of both refractive astigmatism and astigmatism axis subtypes, a finding that aligns with previous reports from China [39,44,46,47], Iran [16], the United States [11], and Mexico [27]. However, other studies have reported a higher prevalence among females [18,21,23] or found no statistically significant differences between the sexes [10,14,17,19,20,23]. Hence, such heterogeneity likely reflects differences in sampling frameworks, age composition, refractive assessment methods, and population-specific environmental or behavioral exposures rather than a strong underlying biological effect of sex.

Although the observed sex-related differences were statistically significant, it is important to contextualize such differences considering their small magnitude of effect (φ_c_ < 0.1), which could indicate that the sex factor accounts for only a limited proportion of the variability in the resulting astigmatism prevalence. From a clinical and epidemiological perspective, this suggests that sex alone is unlikely to be a meaningful determinant of individual risk stratification or targeted screening.

The above is further supported by the multivariable logistic regression analysis adjusted for demographic covariates. While males exhibited higher odds of refractive astigmatism compared with females (aOR = 1.269; 95% CI, 1.265–1.272), the magnitude of this association was relatively modest. Consequently, although sex-related differences are consistently detectable, their clinical and public health relevance may be limited in isolation and should be interpreted within the broader context of sociodemographic, environmental, genetic, and ocular risk factors.

Age-related patterns in the prevalence and distribution of refractive astigmatism, including shifts in axis-associated subtypes, have been extensively documented and reviewed in the literature [2,33,48]. While most literature suggests a steady increase in the prevalence of astigmatism with aging, we observed a non-linear, U-shaped association between age and the prevalence of refractive astigmatism, supported by both descriptive analysis and adjusted multivariable logistic regression modeling. The prevalence increased from childhood and peaked during adolescence (15–19 years; 51.49%), followed by a progressive decline through middle adulthood, stabilizing between 40 and 59 years, with a subsequent increase in older age groups (≥60 years; 36.33–46.02%). Such tendency was mirrored in the logistic regression analysis, in which adolescents exhibited the highest adjusted odds of refractive astigmatism (15–19 years; aOR = 1.47; 95% CI, 1.456–1.484), whereas individuals in midlife (40–59 years) showed substantially lower odds (aORs = 0.580–0.551) before increasing again in older adults (≥70 years; aOR = 1.119–1.117).

Interestingly, the plateau observed between the early 40s and late 50s in both prevalence and adjusted odds coincided with the transitional shift from WTR to ATR astigmatism. Consequently, rather than representing a true reduction in the astigmatism burden, this tendency may reflect a transitional phase characterized by changes in axis orientation and redistribution of refractive components. This interpretation is consistent with longitudinal evidence from Beesley and Elliot [34], Namba et al. [49], and Sanfilippo et al. [33], who collectively described that axis shifts typically emerge during the fourth and fifth decades of life.

Several biological mechanisms may underlie this phenomenon. Age-related corneal biomechanical remodeling, particularly the progressive flattening of the vertical meridian, has been implicated in axial shifts [50]. Alterations in corneal properties, such as elasticity, stiffness, and thickness, all driven by collagen cross-linking, may alter the corneal shape and refractive behavior over time [51,52]. Lenticular factors, including modifications of the refractive index gradient and surface curvature of the crystalline lens, may further contribute to astigmatism variations [53,54]. On the other hand, extrinsic influences, such as reduced eyelid tension with aging, may also affect corneal curvature and axis orientation [55].

Furthermore, methodological or differential healthcare-seeking behavior effects inherent to the study design should also be considered. The use of non-cycloplegic refraction may introduce age-dependent measurement variability, with accommodative effects potentially inflating epidemiological estimates in younger individuals and stabilizing the measurements in middle-aged groups. However, previous studies have reported no statistically significant differences between cycloplegic and non-cycloplegic assessments of refractive astigmatism [56,57,58], suggesting a limited epidemiological impact. In addition, the clinic-based nature of the dataset may reflect differences in healthcare utilization patterns, such as a higher demand for refractive correction in younger individuals (<30 years), reduced utilization during midlife, and increased examinations in older individuals (>60 years) due to visual symptoms, which may partially explain the observed non-linear trend [59,60].

Collectively, these biomechanical and anatomical processes, along with contextual factors, provide a plausible explanation for the middle-life plateau and subsequent increase in astigmatism observed in this study. However, given the clinic-based and retrospective nature of the data, further population-based and longitudinal prospective studies using ocular biometric data, such as corneal curvature and anterior segment parameters, are needed to better characterize these mechanisms in the Mexican population. This will aid in clarifying their clinical implications, particularly concerning axis variations, visual function, and refractive correction strategies.

Regarding geographic distribution, our results indicated that astigmatism is widely distributed across Mexico, although a higher prevalence was concentrated in the central region, particularly in Tlaxcala (57.42%; 95% CI, 56.44–58.41%), Mexico City (52.04%; 95% CI, 51.87–52.21%), and the State of Mexico (51.37%; 95% CI, 51.23–51.51%). This pattern was consistent with the adjusted logistic regression analysis, which also demonstrated significant geographical heterogeneity. Compared with Chiapas, all states showed higher odds of refractive astigmatism, with the largest increases observed in Tlaxcala (aOR = 3.581; 95% CI, 3.491–3.675), Mexico City (aOR = 2.77; 95% CI, 2.74–2.801), and the State of Mexico (aOR = 2.72; 95% CI, 2.69–2.749).

Although the agreement between descriptive and demographic-adjusted findings suggests that these regional differences are not solely attributable to variations in age or sex distribution, the magnitude and consistency of these associations should be interpreted with caution, given the clinic-based nature of the data and the potential influence of unmeasured confounders. For example, the observed geographic variation may reflect, at least in part, differences in eye healthcare access, service utilization, and primary eye care referral patterns, particularly in more urbanized and densely populated areas. Furthermore, additional factors such as genetic predisposition, environmental exposure, behavioral patterns, and regional socioeconomic conditions may also contribute to the observed heterogeneity.

Altogether, these findings highlight that refractive astigmatism is a dynamic condition influenced by a complex interplay of sociodemographic, biological, and contextual factors not analyzed in the present study, which require further exploration. From a clinical and public health perspective, the higher burden observed in adolescents and older adults suggests that these groups may benefit from enhanced screening and access to refractive correction. Similarly, geographic concentration in more urbanized regions may reflect areas of increased service demand, supporting the need for adequate resource allocation and diagnostic capacity in lacking areas. Importantly, given the modest effect sizes and clinic-based nature of the data, these results are insufficient to support targeted screening strategies based on age, sex, or geographic location.

This study provides valuable insights for eye care professionals, clinicians, and policymakers regarding the distribution of refractive astigmatism and its axis-associated subtypes by sex, age group, and region in Mexico. Integrating these epidemiological patterns within a broader context of eye health will provide a foundation for future research and help to optimize screening strategies and guide public health policies aimed at reducing the burden of astigmatism in Mexico.

### Strengths and Limitations

The strengths of this study include its considerably large sample size derived from a wide age range of Mexican individuals, which allowed for a detailed and age-specific analysis of astigmatism patterns in this population. The nationwide multicenter setting and standardized data collection and eye examination protocols, spanning all 32 states of Mexico, warrant broad geographic representation and help to minimize regional bias. Moreover, the use of age-adjusted prevalence estimates, which account for potential biases associated with the age structure of the population, along with multivariable logistic regression models that consider age, sex, and state of residence as confounders, strengthens the validity of the findings.

While a few previous studies have examined the prevalence of refractive astigmatism in Mexico, none to date have assessed its prevalence, age-related dynamics, and geographic distribution of axis-associated subtypes of astigmatism at a comparable scale, a critical gap in ophthalmic epidemiology that this study addresses. Furthermore, by analyzing data from 2021–2023, this study provides comprehensive and up-to-date insights into the burden of astigmatism in Mexico, especially within the post-pandemic context.

However, several limitations should be acknowledged. First, as a retrospective and cross-sectional analysis, it cannot establish causal relationships between the prevalence of refractive astigmatism and sex-specific differences or axis-associated subtypes of astigmatism and the state-level distribution. Therefore, further longitudinal, prospective, and population-based studies are needed to investigate these possible causal relationships, including ocular biometric data, such as corneal curvature and anterior segment parameters, behavioral patterns, genetic predisposition, and environmental or occupational exposure information that may underlie the sex- and region-based distribution of refractive astigmatism in the Mexican population. Second, the exclusive use of electronic health records from individuals who attended Salud Digna clinics introduces potential selection bias and may limit the generalizability of the findings to a broader population.

Third, the use of non-cycloplegic refraction may introduce measurement bias in the epidemiological estimation of refractive errors owing to accommodative effects, especially among children and adolescents. However, cycloplegia primarily affects the spherical component, whereas its impact on cylindrical power and the astigmatism axis remains minimal. Previous research has indicated no statistically significant difference in astigmatism assessment between cycloplegic and non-cycloplegic conditions [56,57,58], suggesting a limited clinical and epidemiological impact. Despite these limitations, this study establishes the first nationwide epidemiological characterization of astigmatism axis subtypes in Mexico, providing valuable insights for future public health planning and laying the foundation for clinical and epidemiological research in the field of visual care in the country.

## 5. Conclusions

Refractive astigmatism was most prevalent among males across both children/adolescent and adult outpatient populations in Mexico, although with modest effect sizes. Distinct age-related patterns were identified, characterized by a non-linear and non-monotonic association with age, a predominance of WTR astigmatism in younger individuals, and a progressive shift towards ATR astigmatism in older adults. An unreported decline in the overall astigmatism prevalence during midlife was also observed, suggesting a possible transient refractive stage that requires further clinical and epidemiological investigation. These findings provide robust real-world evidence from a large nationwide outpatient cohort that enhances the current epidemiological knowledge of astigmatism in Mexico and may support future clinical and epidemiological research and inform public health planning aimed at addressing the burden of astigmatism in the country.

## Figures and Tables

**Table 1:** Overview of distribution and refraction error data among Mexican outpatients.

Characteristic	Study Population		
	General (*n* = 8,622,191)	Female (*n* = 5,466,362)	Male (*n* = 3,155,829)
Age, years, median (IQR)	45 (25–57)	45 (26–57)	44 (24–57)
Refractive error data (D), median (IQR)			
Spherical equivalent (SE)	0.00 D (−1.00–+1.00)	0.00 D (−0.88–+1.12)	−0.12 D (−1.00–+0.75)
Cylinder power (Cyl)	−0.50 D (−1.00–0.00)	−0.50 D (−1.00–0.00)	−0.50 D (−1.25–0.00)
Age groups, *n* (%)			
5–9	233,771 (2.71)	121,187 (2.22)	112,584 (3.57)
10–14	519,985 (6.03)	290,035 (5.31)	229,950 (7.29)
15–19	645,817 (7.49)	397,636 (7.27)	248,181 (7.86)
20–29	1,262,010 (14.64)	795,912 (14.56)	466,098 (14.77)
30–39	1,006,190 (11.67)	643,323 (11.77)	362,867 (11.50)
40–49	1,492,472 (17.31)	991,243 (18.13)	501,229 (15.88)
50–59	1,637,464 (18.99)	1,075,014 (19.67)	562,450 (17.82)
60–69	1,138,852 (13.21)	728,008 (13.32)	410,844 (13.02)
70–79	537,604 (6.24)	332,873 (6.09)	204,731 (6.49)
80–90	148,026 (1.72)	91,131 (1.67)	56,895 (1.80)
Refractive status, *n* (%)			
−0.50 D < SE < +0.50 D	607,900 (18.09)	1,258,712 (23.02)	747,362 (23.68)
SE ≤ −0.50 D	1,130,572 (13.11)	767,758 (14.05)	362,814 (11.50)
SE ≥ +0.50 D	2,124,505 (24.64)	1,436,149 (26.27)	688,356 (21.81)
Cyl ≤ −0.75 D	3,361,040 (38.98)	2,003,743 (36.66)	1,357,297 (43.01)
Astigmatic individuals (Cyl ≤ −0.75 D) with spherical equivalent refraction, *n* (%)			
−0.50 D < SE < +0.50 D	607,900 (18.09)	363,971 (18.16)	243,929 (17.97)
SE ≤ −0.50 D	2,040,822 (60.72)	1,188,703 (59.32)	852,119 (62.78)
SE ≥ +0.50 D	712,318 (21.19)	451,069 (22.51)	261,249 (19.25)
Astigmatism axis subtypes, *n* (%)			
With-the-rule (WTR)	2,261,141 (67.28)	1,348,048 (67.28)	913,093 (67.27)
Against-the-rule (ATR)	880,965 (26.21)	518,971 (25.90)	361,994 (26.67)
Oblique (OBL)	218,934 (6.51)	136,724 (6.82)	82,210 (6.06)

D, diopter; IQR, interquartile range.

**Table 2:** Age group- and sex-specific crude prevalence estimates of refractive astigmatism among Mexican outpatients.

Age (Years)	Total Individuals (*n*)	Refractive Astigmatism (Cyl ≤ −0.75 D)										
		General		Female			Male			*χ^2^*	*p*-Value	φ_c_
		n	% (95% CI)	Total	n	% (95% CI)	Total	n	% (95% CI)			
5–9	233,771	101,337	43.35 (43.15–43.55)	121,187	50,572	41.73 (41.45–42.01)	112,584	50,765	45.09 (44.8–45.38)	268.34	**<0.001**	**<0.1**
10–14	519,985	253,977	48.84 (48.71–48.98)	290,035	135,470	46.71 (46.53–46.89)	229,950	118,507	51.54 (51.33–51.74)	1196.44	**<0.001**	**<0.1**
15–19	645,817	332,541	51.49 (51.37–51.61)	397,636	188,948	47.52 (47.36–47.67)	248,181	143,593	57.86 (57.66–58.05)	6541.26	**<0.001**	**0.1**
20–29	1,262,010	609,073	48.26 (48.17–48.35)	795,912	359,388	45.15 (45.04–45.26)	466,098	249,685	53.57 (53.43–53.71)	8336.23	**<0.001**	**<0.1**
30–39	1,006,190	443,483	44.08 (43.98–44.17)	643,323	265,437	41.26 (41.14–41.38)	362,867	178,046	49.07 (48.9–49.23)	5735.51	**<0.001**	**<0.1**
40–49	1,492,472	434,660	29.12 (29.05–29.20)	991,243	271,194	27.36 (27.27–27.45)	501,229	163,466	32.61 (32.48–32.74)	4451.98	**<0.001**	**<0.1**
50–59	1,637,464	463,685	28.32 (28.25–28.39)	1,075,014	293,072	27.26 (27.18–27.35)	562,450	170,613	30.33 (30.21–30.45)	1716.44	**<0.001**	**<0.1**
60–69	1,138,852	413,713	36.33 (36.24–36.42)	728,008	254,697	34.99 (34.88–35.1)	410,844	159,016	38.7 (38.56–38.85)	1570.62	**<0.001**	**<0.1**
70–79	537,604	240,446	44.73 (44.59–44.86)	332,873	143,923	43.24 (43.07–43.41)	204,731	96,523	47.15 (46.93–47.36)	783.78	**<0.001**	**<0.1**
80–90	148,026	68,125	46.02 (45.77–46.28)	91,131	41,042	45.04 (44.71–45.36)	56,895	27,083	47.6 (47.19–48.01)	92.80	**<0.001**	**<0.1**

CI, confidence interval; D, diopter. The Chi-square test of independence was used to test the association between sex and the prevalence of refractive astigmatism. The effect size was estimated using Cramér’s *V* coefficient (φ_c_). A *p*-value < 0.05 was considered statistically significant; statistically significant values are shown in bold.

**Table 3:** Univariable and multivariable logistic regression analyses of refractive astigmatism among Mexican outpatients.

Variables	Univariable		Multivariable	
	cOR ^a^ (95% CI)	*p*-Value	aOR ^b^ (95% CI)	*p*-Value
Age groups				
5–9 (Ref)	1.000	—	1.000	—
10–14	1.248 (1.236–1.260)	**<0.001**	1.283 (1.27–1.295)	**<0.001**
15–19	1.387 (1.374–1.401)	**<0.001**	1.47 (1.456–1.484)	**<0.001**
20–29	1.219 (1.208–1.230)	**<0.001**	1.299 (1.288–1.311)	**<0.001**
30–39	1.030 (1.021–1.039)	**<0.001**	1.088 (1.078–1.098)	**<0.001**
40–49	0.537 (0.532–0.542)	**<0.001**	0.58 (0.575–0.585)	**<0.001**
50–59	0.516 (0.512–0.521)	**<0.001**	0.551 (0.546–0.556)	**<0.001**
60–69	0.746 (0.739–0.752)	**<0.001**	0.796 (0.789–0.803)	**<0.001**
70–79	1.057 (1.047–1.068)	**<0.001**	1.119 (1.108–1.13)	**<0.001**
80–90	1.114 (1.100–1.129)	**<0.001**	1.177 (1.161–1.193)	**<0.001**
Sex				
Female (Ref)	1.000	—	1.000	—
Male	1.304 (1.300–1.308)	**<0.001**	1.269 (1.265–1.272)	**<0.001**
State of residence				
Chiapas (Ref)	1.000	—	1.000	—
Aguascalientes	1.853 (1.826–1.882)	**<0.001**	1.817 (1.79–1.846)	**<0.001**
Baja California	1.652 (1.633–1.671)	**<0.001**	1.636 (1.617–1.655)	**<0.001**
Baja California Sur	1.663 (1.623–1.703)	**<0.001**	1.687 (1.646–1.728)	**<0.001**
Campeche	1.690 (1.647–1.734)	**<0.001**	1.712 (1.668–1.758)	**<0.001**
Chihuahua	1.733 (1.710–1.756)	**<0.001**	1.766 (1.742–1.79)	**<0.001**
Mexico City	2.716 (2.687–2.746)	**<0.001**	2.77 (2.74–2.801)	**<0.001**
Coahuila	1.764 (1.740–1.789)	**<0.001**	1.768 (1.743–1.793)	**<0.001**
Colima	1.155 (1.133–1.178)	**<0.001**	1.178 (1.156–1.202)	**<0.001**
Durango	1.463 (1.442–1.485)	**<0.001**	1.461 (1.439–1.484)	**<0.001**
State of Mexico	2.742 (2.713–2.772)	**<0.001**	2.72 (2.69–2.749)	**<0.001**
Guanajuato	1.889 (1.867–1.911)	**<0.001**	1.885 (1.863–1.908)	**<0.001**
Guerrero	1.242 (1.220–1.264)	**<0.001**	1.322 (1.299–1.346)	**<0.001**
Hidalgo	2.009 (1.972–2.048)	**<0.001**	2.013 (1.974–2.052)	**<0.001**
Jalisco	1.661 (1.642–1.680)	**<0.001**	1.649 (1.63–1.668)	**<0.001**
Michoacan	1.392 (1.373–1.412)	**<0.001**	1.39 (1.37–1.409)	**<0.001**
Morelos	1.483 (1.454–1.512)	**<0.001**	1.525 (1.495–1.556)	**<0.001**
Nayarit	1.116 (1.098–1.136)	**<0.001**	1.136 (1.116–1.156)	**<0.001**
Nuevo Leon	1.472 (1.455–1.489)	**<0.001**	1.493 (1.476–1.511)	**<0.001**
Oaxaca	1.294 (1.270–1.319)	**<0.001**	1.299 (1.274–1.325)	**<0.001**
Puebla	2.389 (2.360–2.418)	**<0.001**	2.356 (2.327–2.385)	**<0.001**
Queretaro	2.346 (2.316–2.377)	**<0.001**	2.319 (2.289–2.35)	**<0.001**
Quintana Roo	1.564 (1.537–1.591)	**<0.001**	1.61 (1.583–1.638)	**<0.001**
San Luis Potosi	1.707 (1.682–1.733)	**<0.001**	1.673 (1.648–1.698)	**<0.001**
Sinaloa	1.270 (1.255–1.286)	**<0.001**	1.296 (1.28–1.311)	**<0.001**
Sonora	1.400 (1.383–1.417)	**<0.001**	1.405 (1.388–1.423)	**<0.001**
Tabasco	1.228 (1.208–1.249)	**<0.001**	1.292 (1.27–1.314)	**<0.001**
Tamaulipas	1.263 (1.246–1.281)	**<0.001**	1.338 (1.32–1.357)	**<0.001**
Tlaxcala	3.525 (3.247–3.615)	**<0.001**	3.581 (3.491–3.675)	**<0.001**
Veracruz	1.554 (1.536–1.573)	**<0.001**	1.585 (1.566–1.604)	**<0.001**
Yucatan	1.655 (1.629–1.681)	**<0.001**	1.723 (1.696–1.751)	**<0.001**
Zacatecas	2.378 (2.333–2.423)	**<0.001**	2.362 (2.316–2.407)	**<0.001**

aOR, adjusted odds ratio; cOR, crude odds ratio; CI, confidence interval; Ref, reference. A *p*-value < 0.05 was considered statistically significant; statistically significant values are shown in bold. ^a^ cOR was estimated by univariable logistic regression. ^b^ aOR was estimated by multivariable logistic regression modeling adjusted for age, sex and state of residence.

## Data Availability

The data presented in this study are available on request from the corresponding author due to privacy restrictions established by the Mexican Federal Law on the Protection of Personal Data.

## Supplementary Materials

The following supporting information can be downloaded at: https://www.mdpi.com/article/10.3390/jcm15093522/s1, Table S1: Crude prevalence estimates of refractive astigmatism and axis-associated subtypes among Mexican outpatients; Table S2: Age group- and sex-specific crude prevalence estimates of astigmatism axis subtypes among Mexican outpatients; Table S3: Crude and age-adjusted state-level prevalence estimates of refractive astigmatism among Mexican outpatients; Table S4: Crude and age-adjusted state-level prevalence estimates of with-the-rule (WTR) astigmatism among Mexican outpatients; Table S5: Crude and age-adjusted state-level prevalence estimates of against-the-rule (ATR) astigmatism among Mexican outpatients; Table S6: Crude and age-adjusted state-level prevalence estimates of oblique (OBL) astigmatism among Mexican outpatients.

## Abbreviations

The following abbreviations are used in this manuscript:

aOR	Adjusted odds ratio
cOR	Crude odds ratio
ATR	Against-the-rule astigmatism subtype
CI	Confidence interval
Cyl	Cylinder power
D	Diopter
EHR	Electronic health record
IQR	Interquartile range
OBL	Oblique astigmatism subtype
SE	Spherical equivalent
STROBE	Strengthening the Reporting of Observational Studies in Epidemiology
WHO	World Health Organization
WTR	With-the-rule astigmatism subtype
