# Supplementary material for: Prevalence and Distribution of Axis Subtypes of Refractive Astigmatism in Mexican Outpatients: A Nationwide Multicenter Clinic-Based Cross-Sectional Study
Source: J Clin Med. 2026 May 5;15(9):3522. doi: 10.3390/jcm15093522 (PMC13165337; doi:10.3390/jcm15093522)
Supplement: Supplementary file 1 [file jcm-15-03522-s001.zip › jcm-4248471-supplementary.pdf]

Supplementary Material

Prevalence and distribution of axis subtypes of refractive astigmatism in Mexican outpatients: A nationwide multicenter clinic-based cross-sectional study

José Antonio Magaña-Lizárraga[1]; Abraham García-Gil[1]; Ricardo Daniel Contreras-Espinoza[2]; Eduardo Espinoza-Angulo[2]; Héctor Machado-Jiménez[2]; Marco Antonio Luna-Ruiz-Esparza[1]; Humberto Gómez-Campaña[3]; Abraham Campos-Romero[1]; Jonathan Alcántar-Fernández[1*]

[1] Innovation and Research Department, Salud Digna, Culiacan 80184, Sinaloa, Mexico.
[2] Optometry Department, Salud Digna, Culiacan 80184, Sinaloa, Mexico.
[3] Medical Direction, Salud Digna, Culiacan 80184, Sinaloa, Mexico.

Table S1. Crude prevalence estimates of refractive astigmatism and axis-associated subtypes among Mexican outpatients

Table S2. Age group- and sex-specific crude prevalence estimates of astigmatism axis subtypes among Mexican outpatients

Table S3. Crude and age-adjusted state-level prevalence estimates of refractive astigmatism among Mexican outpatients

Table S4. Crude and age-adjusted state-level prevalence estimates of with-the-rule (WTR) astigmatism among Mexican outpatients

Table S5. Crude and age-adjusted state-level prevalence estimates of against-the-rule (ATR) astigmatism among Mexican outpatients

Table S6. Crude and age-adjusted state-level prevalence estimates of oblique (OBL) astigmatism among Mexican outpatients

Table S1. Crude prevalence estimates of refractive astigmatism and axis-associated subtypes among Mexican outpatients

Age (years)	Total individuals (*n*)	Refractive astigmatism (Cyl ≤ -0.75 D)		With-the-rule (WTR)		Against-the-rule (ATR)		Oblique (OBL)	
		n	% (95% CI)	*n*	% (95% CI)	*n*	% (95% CI)	*n*	% (95% CI)
5-9	233,771	101,337	43.35 (43.15–43.55)	97,935	41.89 (41.69–42.09)	1,803	0.77 (0.74–0.81)	1,599	0.68 (0.65–0.72)
10-14	519,985	253,977	48.84 (48.71–48.98)	243,647	46.86 (46.72–46.99)	5,637	1.08 (1.06–1.11)	4,693	0.9 (0.88–0.93)
15-19	645,817	332,541	51.49 (51.37–51.61)	316,237	48.97 (48.84–49.09)	8,761	1.36 (1.33–1.39)	7,543	1.17 (1.14–1.19)
20-29	1,262,010	609,073	48.26 (48.17–48.35)	553,901	43.89 (43.8–43.98)	31,587	2.5 (2.48–2.53)	23,585	1.87 (1.85–1.89)
30-39	1,006,190	443,483	44.08 (43.98–44.17)	366,165	36.39 (36.3–36.49)	49,354	4.91 (4.86–4.95)	27,964	2.78 (2.75–2.81)
40-49	1,492,472	434,660	29.12 (29.05–29.20)	291,014	19.5 (19.44–19.56)	104,651	7.01 (6.97–7.05)	38,995	2.61 (2.59–2.64)
50-59	1,637,464	463,685	28.32 (28.25–28.39)	219,914	13.43 (13.38–13.48)	197,649	12.07 (12.02–12.12)	46,122	2.82 (2.79–2.84)
60-69	1,138,852	413,713	36.33 (36.24–36.42)	117,985	10.36 (10.3–10.42)	256,413	22.52 (22.44–22.59)	39,315	3.45 (3.42–3.49)
70-79	537,604	240,446	44.73 (44.59–44.86)	45,090	8.39 (8.31–8.46)	172,465	32.08 (31.96–32.21)	22,891	4.26 (4.2–4.31)
80-90	148,026	68,125	46.02 (45.77–46.28)	9,253	6.25 (6.13–6.38)	52,645	35.56 (35.32–35.81)	6,227	4.21 (4.1–4.31)

CI, confidence interval; Cyl, cylinder power; D, diopter.

Table S2. Age group- and sex-specific crude prevalence estimates of astigmatism axis subtypes among Mexican outpatients

Age (years)	With-the-rule (WTR) astigmatism								
	Female			Male			χ^2	*p*-value	φ_c[a]
	Total	*n*	% (95% CI)	Total	*n*	% (95% CI)			
5-9	121,187	48,801	40.27 (39.99–40.55)	112,584	49,134	43.64 (43.35–43.93)	6.53	**0.01**	< 0.1
10-14	290,035	129,894	44.79 (44.6–44.97)	229,950	113,753	49.47 (49.26–49.67)	1.77	0.18	< 0.1
15-19	397,636	179,466	45.13 (44.98–45.29)	248,181	136,771	55.11 (54.91–55.31)	12.51	**< 0.001**	< 0.1
20-29	795,912	328,491	41.27 (41.16–41.38)	466,098	225,410	48.36 (48.22–48.5)	226.40	**< 0.001**	< 0.1
30-39	643,323	221,399	34.41 (34.3–34.53)	362,867	144,766	39.9 (39.74–40.05)	326.80	**< 0.001**	< 0.1
40-49	991,243	184,291	18.59 (18.52–18.67)	501,229	106,723	21.29 (21.18–21.41)	328.07	**< 0.001**	< 0.1
50-59	1,075,014	143,179	13.32 (13.25–13.38)	562,450	76,735	13.64 (13.55–13.73)	650.58	**< 0.001**	< 0.1
60-69	728,008	77,253	10.61 (10.54–10.68)	410,844	40,732	9.91 (9.82–10.01)	1,068.19	**< 0.001**	< 0.1
70-79	332,873	29,227	8.78 (8.68–8.88)	204,731	15,863	7.75 (7.63–7.86)	568.80	**< 0.001**	< 0.1
80-90	91,131	6,047	6.64 (6.47–6.8)	56,895	3,206	5.63 (5.45–5.83)	116.58	**< 0.001**	< 0.1

Age (years)	Against-the-rule (ATR) astigmatism								
	Female			Male			χ^2	*p*-value	φ_c[a]
	Total	*n*	% (95% CI)	Total	*n*	% (95% CI)			
5-9	121,187	1,001	0.83 (0.78–0.88)	112,584	802	0.71 (0.66–0.76)	23.14	**< 0.001**	< 0.1
10-14	290,035	3,158	1.09 (1.05–1.13)	229,950	2,479	1.08 (1.04–1.12)	16.68	**< 0.001**	< 0.1
15-19	397,636	5,241	1.32 (1.28–1.35)	248,181	3,520	1.42 (1.37–1.47)	33.06	**< 0.001**	< 0.1
20-29	795,912	17,513	2.2 (2.17–2.23)	466,098	14,074	3.02 (2.97–3.07)	174.75	**< 0.001**	< 0.1
30-39	643,323	27,262	4.24 (4.19–4.29)	362,867	22,092	6.09 (6.01–6.17)	492.25	**< 0.001**	< 0.1
40-49	991,243	62,005	6.26 (6.21–6.3)	501,229	42,646	8.51 (8.43–8.59)	580.26	**< 0.001**	< 0.1
50-59	1,075,014	119,796	11.14 (11.08–11.2)	562,450	77,853	13.84 (13.75–13.93)	997.11	**< 0.001**	< 0.1
60-69	728,008	152,069	20.89 (20.8–20.98)	410,844	104,344	25.4 (25.26–25.53)	1,452.34	**< 0.001**	< 0.1
70-79	332,873	99,961	30.03 (29.87–30.19)	204,731	72,504	35.41 (35.21–35.62)	913.10	**< 0.001**	< 0.1
80-90	91,131	30,965	33.98 (33.67–34.29)	56,895	21,680	38.11 (37.71–38.51)	196.88	**< 0.001**	< 0.1

Age (years)	Oblique (OBL) astigmatism								
	Female			Male			χ^2	*p*-value	φ_c[a]
	Total	*n*	% (95% CI)	Total	*n*	% (95% CI)			
5-9	121,187	770	0.64 (0.59–0.68)	112,584	829	0.74 (0.69–0.79)	1.99	0.16	< 0.1
10-14	290,035	2,418	0.83 (0.8–0.87)	229,950	2,275	0.99 (0.95–1.03)	6.34	**0.01**	< 0.1
15-19	397,636	4,241	1.07 (1.03–1.1)	248,181	3,302	1.33 (1.29–1.38)	1.11	0.29	< 0.1
20-29	795,912	13,384	1.68 (1.65–1.71)	466,098	10,201	2.19 (2.15–2.23)	51.71	**< 0.001**	< 0.1
30-39	643,323	16,776	2.61 (2.57–2.65)	362,867	11,188	3.08 (3.03–3.14)	0.24	0.63	< 0.1
40-49	991,243	24,898	2.51 (2.48–2.54)	501,229	14,097	2.81 (2.77–2.86)	38.76	**< 0.001**	< 0.1
50-59	1,075,014	30,097	2.8 (2.77–2.83)	562,450	16,025	2.85 (2.81–2.89)	92.57	**< 0.001**	< 0.1
60-69	728,008	25,375	3.49 (3.44–3.53)	410,844	13,940	3.39 (3.34–3.45)	162.94	**< 0.001**	< 0.1
70-79	332,873	14,735	4.43 (4.36–4.5)	204,731	8,156	3.98 (3.9–4.07)	214.50	**< 0.001**	< 0.1
80-90	91,131	4,030	4.42 (4.29–4.56)	56,895	2,197	3.86 (3.7–4.02)	57.25	**< 0.001**	< 0.1

CI, confidence interval. The Chi-square test of independence was used to test the association between sex and the prevalence of each astigmatism axis subtype. Effect size was estimated using Cramér's *V* coefficient (φ_c). A *p*-value < 0.05 was considered statistically significant; statistically significant values are shown in bold.

[a] Cramér's *V* coefficient (φ_c)

Table S3. Crude and age-adjusted state-level prevalence estimates of refractive astigmatism among Mexican outpatients

State	Total individuals (***n***)	Refractive astigmatism (Cyl ≤ -0.75 D)													
		General			Female				Male						
		n	Crude % (95% CI)	Age-adjusted[a] % (95% CI)	Total	*n*	Crude % (95% CI)	Age-adjusted[a] % (95% CI)	Total	*n*	Crude % (95% CI)	Age-adjusted[a] % (95% CI)	χ^2	*p*-value	φ_c[b]
Aguascalientes	127,088	49,515	38.96 (38.69–39.23)	40.21 (39.82–40.6)	80,191	29,640	36.96 (36.63–37.3)	38.33 (37.83–38.83)	46,897	19,875	42.38 (41.93–42.83)	43.37 (42.73–44.02)	365.31	**< 0.001**	< 0.1
Baja California	573,401	207,948	36.27 (36.14–36.39)	38.19 (38.01–38.37)	357,516	121,779	34.06 (33.91–34.22)	36.04 (35.81–36.27)	215,885	86,169	39.91 (39.71–40.12)	41.78 (41.48–42.09)	1,994.18	**< 0.001**	< 0.1
Baja California Sur	34,902	12,708	36.41 (35.91–36.92)	37.77 (37.01–38.54)	22,662	7,617	33.61 (33–34.23)	35.29 (34.35–36.27)	12,240	5,091	41.59 (40.72–42.47)	42.56 (41.26–43.91)	218.69	**< 0.001**	< 0.1
Campeche	29,267	10,766	36.79 (36.23–37.34)	38.75 (37.9–39.61)	18,048	6,143	34.04 (33.35–34.73)	36.04 (34.96–37.17)	11,219	4,623	41.21 (40.29–42.12)	43.03 (41.66–44.44)	152.94	**< 0.001**	< 0.1
Chiapas	197,530	50,601	25.62 (25.42–25.81)	27.84 (27.55–28.13)	123,075	28,802	23.4 (23.17–23.64)	25.85 (25.48–26.22)	74,455	21,799	29.28 (28.95–29.61)	31.19 (30.73–31.66)	840.64	**< 0.001**	< 0.1
Chihuahua	209,538	78,313	37.37 (37.17–37.58)	40.05 (39.74–40.37)	134,995	47,974	35.54 (35.28–35.79)	38.23 (37.84–38.63)	74,543	30,339	40.7 (40.35–41.05)	43.18 (42.65–43.71)	546.81	**< 0.001**	< 0.1
Mexico City	961,200	464,590	48.33 (48.23–48.43)	52.04 (51.87–52.21)	611,102	282,439	46.22 (46.09–46.34)	50.05 (49.83–50.27)	350,098	182,151	52.03 (51.86–52.19)	55.28 (55.01–55.56)	3,009.35	**< 0.001**	< 0.1
Coahuila	180,639	68,278	37.8 (37.57–38.02)	41.16 (40.81–41.51)	116,404	41,754	35.87 (35.59–36.15)	39.37 (38.93–39.81)	64,235	26,524	41.29 (40.91–41.67)	44.3 (43.72–44.89)	517.62	**< 0.001**	< 0.1
Colima	69,345	19,735	28.46 (28.12–28.8)	28.71 (28.22–29.2)	43,308	11,426	26.38 (25.97–26.8)	26.56 (25.96–27.18)	26,037	8,309	31.91 (31.35–32.48)	32.28 (31.47–33.11)	244.17	**< 0.001**	< 0.1
Durango	144,636	48,462	33.51 (33.26–33.75)	36.66 (36.3–37.02)	94,491	29,598	31.32 (31.03–31.62)	34.77 (34.32–35.22)	50,145	18,864	37.62 (37.19–38.04)	40.04 (39.44–40.66)	582.73	**< 0.001**	< 0.1
State of Mexico	1,358,039	659,610	48.57 (48.49–48.65)	51.37 (51.23–51.5)	843,776	390,158	46.24 (46.13–46.35)	49.18 (49.01–49.35)	514,263	269,452	52.4 (52.26–52.53)	54.74 (54.52–54.95)	4,847.81	**< 0.001**	< 0.1
Guanajuato	435,611	171,698	39.42 (39.27–39.56)	41.76 (41.55–41.98)	271,978	100,308	36.88 (36.7–37.06)	39.46 (39.18–39.74)	163,633	71,390	43.63 (43.39–43.87)	45.4 (45.04–45.76)	1,947.71	**< 0.001**	< 0.1
Guerrero	85,456	25,602	29.96 (29.65–30.27)	30.95 (30.46–31.45)	56,684	15,767	27.82 (27.45–28.19)	28.55 (27.95–29.18)	28,772	9,835	34.18 (33.63–34.73)	35.51 (34.67–36.37)	368.69	**< 0.001**	< 0.1
Hidalgo	62,559	25,585	40.9 (40.51–41.28)	44.36 (43.76–44.97)	39,010	14,924	38.26 (37.77–38.74)	41.84 (41.07–42.62)	23,549	10,661	45.27 (44.63–45.91)	48.38 (47.4–49.38)	298.93	**< 0.001**	< 0.1
Jalisco	647,682	235,669	36.39 (36.27–36.5)	37.79 (37.62–37.96)	408,946	140,400	34.33 (34.19–34.48)	35.71 (35.5–35.93)	238,736	95,269	39.91 (39.71–40.1)	41.17 (40.88–41.45)	2,022.91	**< 0.001**	< 0.1
Michoacan	185,448	60,092	32.4 (32.19–32.62)	34.98 (34.66–35.3)	119,112	36,024	30.24 (29.98–30.51)	32.99 (32.59–33.4)	66,336	24,068	36.28 (35.92–36.65)	38.42 (37.89–38.96)	709.21	**< 0.001**	< 0.1
Morelos	59,880	20,242	33.8 (33.43–34.18)	35.65 (35.07–36.26)	38,987	12,352	31.68 (31.22–32.15)	33.46 (32.72–34.23)	20,893	7,890	37.76 (37.11–38.43)	39.37 (38.39–40.37)	224.83	**< 0.001**	< 0.1
Nayarit	101,399	28,159	27.77 (27.49–28.05)	30.55 (30.13–30.97)	67,250	17,227	25.62 (25.29–25.95)	28.31 (27.8–28.83)	34,149	10,932	32.01 (31.52–32.51)	34.91 (34.17–35.67)	461.95	**< 0.001**	< 0.1
Nuevo Leon	534,527	179,828	33.64 (33.52–33.77)	36.38 (36.19–36.58)	339,820	107,683	31.69 (31.53–31.84)	34.4 (34.15–34.65)	194,707	72,145	37.05 (36.84–37.27)	39.75 (39.43–40.07)	1,595.88	**< 0.001**	< 0.1
Oaxaca	67,160	20,706	30.83 (30.48–31.18)	34.06 (33.53–34.6)	43,080	12,356	28.68 (28.25–29.11)	32.45 (31.77–33.16)	24,080	8,350	34.68 (34.07–35.28)	36.88 (36.03–37.76)	260.28	**< 0.001**	< 0.1
Puebla	354,046	159,813	45.14 (44.98–45.3)	47.35 (47.09–47.62)	219,737	93,337	42.48 (42.27–42.68)	44.94 (44.6–45.28)	134,309	66,476	49.49 (49.23–49.76)	51.15 (50.73–51.58)	1,657.97	**< 0.001**	< 0.1
Queretaro	230,140	102,844	44.69 (44.48–44.89)	46.36 (46.04–46.69)	140,355	59,046	42.07 (41.81–42.33)	43.97 (43.56–44.39)	89,785	43,798	48.78 (48.45–49.11)	49.99 (49.48–50.5)	997.99	**< 0.001**	< 0.1
Quintana Roo	90,541	31,692	35 (34.69–35.31)	36.75 (36.27–37.25)	57,723	18,617	32.25 (31.87–32.64)	34.12 (33.52–34.74)	32,818	13,075	39.84 (39.31–40.37)	41.24 (40.44–42.07)	529.60	**< 0.001**	< 0.1
San Luis Potosi	138,236	51,183	37.03 (36.77–37.28)	40.37 (39.99–40.76)	88,682	30,900	34.84 (34.53–35.16)	38.38 (37.9–38.87)	49,554	20,283	40.93 (40.5–41.37)	43.87 (43.24–44.52)	505.25	**< 0.001**	< 0.1
Sinaloa	470,240	143,116	30.43 (30.3–30.57)	31.14 (30.95–31.33)	306,701	86,629	28.25 (28.09–28.41)	29.16 (28.93–29.4)	163,539	56,487	34.54 (34.31–34.77)	35.03 (34.69–35.36)	1,996.37	**< 0.001**	< 0.1
Sonora	356,610	115,989	32.53 (32.37–32.68)	35.72 (35.48–35.95)	228,870	69,496	30.36 (30.18–30.55)	33.63 (33.34–33.93)	127,740	46,493	36.4 (36.13–36.66)	39.48 (39.08–39.87)	1,359.08	**< 0.001**	< 0.1
Tabasco	102,333	30,423	29.73 (29.45–30.01)	31.13 (30.71–31.56)	67,646	18,436	27.25 (26.92–27.59)	28.81 (28.28–29.35)	34,687	11,987	34.56 (34.06–35.06)	35.43 (34.71–36.16)	585.53	**< 0.001**	< 0.1
Tamaulipas	214,611	65,069	30.32 (30.13–30.51)	34.5 (34.18–34.83)	142,205	39,925	28.08 (27.84–28.31)	32.17 (31.77–32.58)	72,406	25,144	34.73 (34.38–35.07)	38.83 (38.27–39.39)	1,004.49	**< 0.001**	< 0.1
Tlaxcala	29,111	15,962	54.83 (54.26–55.4)	57.42 (56.44–58.41)	18,169	9,543	52.52 (51.79–53.25)	55.44 (54.18–56.74)	10,942	6,419	58.66 (57.73–59.59)	60.57 (59–62.18)	103.97	**< 0.001**	< 0.1
Veracruz	398,163	138,822	34.87 (34.72–35.01)	35.75 (35.53–35.97)	255,147	82,658	32.4 (32.21–32.58)	33.22 (32.95–33.49)	143,016	56,164	39.27 (39.02–39.52)	40.13 (39.76–40.5)	1,907.39	**< 0.001**	< 0.1
Yucatan	112,344	40,778	36.3 (36.02–36.58)	37.25 (36.82–37.69)	72,688	24,443	33.63 (33.28–33.97)	34.33 (33.79–34.89)	39,656	16,335	41.19 (40.71–41.68)	42.14 (41.41–42.88)	634.96	**< 0.001**	< 0.1
Zacatecas	60,509	27,242	45.02 (44.62–45.42)	47.19 (46.58–47.8)	38,004	16,342	43 (42.5–43.5)	45.35 (44.57–46.14)	22,505	10,900	48.43 (47.78–49.09)	50.19 (49.2–51.2)	168.56	**< 0.001**	< 0.1

CI, confidence interval; Cyl, cylinder power; D, diopter. The Chi-square test of independence was used to test the association between sex and the prevalence of refractive astigmatism. Effect size was estimated using Cramér's *V* coefficient (φ_c). A *p*-value < 0.05 was considered statistically significant; statistically significant values are shown in bold.

[a]Age-adjusted prevalence to the World Health Organization (WHO) world standard population.

[b]Cramér's *V* coefficient (φ_c)

Table S4. Crude and age-adjusted state-level prevalence estimates of with-the-rule (WTR) astigmatism among Mexican outpatients

State	Total individuals (*n*)	With-the-rule (WTR) astigmatism													
		General			Female				Male						
		n	Crude % (95% CI)	Age-adjusted[a] % (95% CI)	Total	*n*	Crude % (95% CI)	Age-adjusted[a] % (95% CI)	Total	*n*	Crude % (95% CI)	Age-adjusted[a] % (95% CI)	X^2	*p*-value	φ_c[b]
Aguascalientes	127,088	33,586	26.43 (26.19–26.67)	30.19 (29.84–30.55)	80,191	20,406	25.45 (25.15–25.75)	29.19 (28.73–29.65)	46,897	13,180	28.1 (27.7–28.51)	31.83 (31.26–32.42)	34.95	**< 0.001**	< 0.1
Baja California	573,401	133,489	23.28 (23.17–23.39)	27.69 (27.52–27.85)	357,516	78,686	22.01 (21.87–22.15)	26.25 (26.04–26.46)	215,885	54,803	25.39 (25.2–25.57)	30.1 (29.83–30.37)	22.59	**< 0.001**	< 0.1
Baja California Sur	34,902	7,522	21.55 (21.12–21.99)	26.46 (25.79–27.16)	22,662	4,609	20.34 (19.82–20.87)	25.04 (24.2–25.93)	12,240	2,913	23.8 (23.05–24.56)	29.26 (28.12–30.46)	13.68	**< 0.001**	< 0.1
Campeche	29,267	6,276	21.44 (20.97–21.92)	27.44 (26.67–28.22)	18,048	3,508	19.44 (18.86–20.02)	25.31 (24.32–26.34)	11,219	2,768	24.67 (23.88–25.48)	30.67 (29.45–31.95)	8.32	**0.004**	< 0.1
Chiapas	197,530	30,241	15.31 (15.15–15.47)	20.05 (19.79–20.31)	123,075	17,237	14.01 (13.81–14.2)	18.72 (18.38–19.06)	74,455	13,004	17.47 (17.19–17.74)	22.31 (21.89–22.73)	0.19	0.66	< 0.1
Chihuahua	209,538	50,018	23.87 (23.69–24.05)	29.95 (29.67–30.24)	134,995	30,878	22.87 (22.65–23.1)	28.99 (28.63–29.36)	74,543	19,140	25.68 (25.36–25.99)	31.53 (31.06–32.01)	13.13	**< 0.001**	< 0.1
Mexico City	961,200	334,474	34.8 (34.7–34.89)	42.6 (42.44–42.76)	611,102	202,903	33.2 (33.08–33.32)	41.24 (41.04–41.45)	350,098	131,571	37.58 (37.42–37.74)	44.67 (44.41–44.92)	8.45	**0.004**	< 0.1
Coahuila	180,639	47,914	26.52 (26.32–26.73)	32.19 (31.87–32.51)	116,404	29,590	25.42 (25.17–25.67)	31.05 (30.65–31.46)	64,235	18,324	28.53 (28.18–28.88)	34.13 (33.61–34.67)	24.63	**< 0.001**	< 0.1
Colima	69,345	10,010	14.44 (14.17–14.7)	19.36 (18.92–19.8)	43,308	5,775	13.33 (13.02–13.66)	17.9 (17.36–18.47)	26,037	4,235	16.27 (15.82–16.72)	21.71 (20.99–22.46)	0.35	0.55	< 0.1
Durango	144,636	33,680	23.29 (23.07–23.5)	28.66 (28.33–28.99)	94,491	20,861	22.08 (21.81–22.34)	27.53 (27.11–27.95)	50,145	12,819	25.56 (25.18–25.95)	30.62 (30.07–31.18)	34.69	**< 0.001**	< 0.1
State of Mexico	1,358,039	499,560	36.79 (36.7–36.87)	42 (41.88–42.13)	843,776	294,938	34.95 (34.85–35.06)	40.41 (40.25–40.57)	514,263	204,622	39.79 (39.66–39.92)	44.33 (44.13–44.53)	10.36	**0.001**	< 0.1
Guanajuato	435,611	120,603	27.69 (27.55–27.82)	32.52 (32.32–32.72)	271,978	70,929	26.08 (25.91–26.24)	31.09 (30.83–31.35)	163,633	49,674	30.36 (30.13–30.58)	34.66 (34.34–34.99)	25.48	**< 0.001**	< 0.1
Guerrero	85,456	11,165	13.07 (12.84–13.29)	19.63 (19.2–20.08)	56,684	6,588	11.62 (11.36–11.89)	17.78 (17.24–18.35)	28,772	4,577	15.91 (15.49–16.34)	23.02 (22.28–23.78)	55.67	**< 0.001**	< 0.1
Hidalgo	62,559	19,335	30.91 (30.54–31.27)	36.82 (36.25–37.39)	39,010	11,308	28.99 (28.54–29.44)	34.91 (34.18–35.65)	23,549	8,027	34.09 (33.48–34.7)	39.77 (38.85–40.7)	0.77	0.38	< 0.1
Jalisco	647,682	153,321	23.67 (23.57–23.78)	27.99 (27.83–28.15)	408,946	92,686	22.66 (22.54–22.79)	26.85 (26.65–27.05)	238,736	60,635	25.4 (25.22–25.57)	29.74 (29.49–30)	140.19	**< 0.001**	< 0.1
Michoacan	185,448	39,214	21.15 (20.96–21.33)	26.7 (26.41–27)	119,112	23,615	19.83 (19.6–20.05)	25.35 (24.98–25.73)	66,336	15,599	23.52 (23.19–23.84)	28.99 (28.51–29.48)	3.50	0.06	< 0.1
Morelos	59,880	12,054	20.13 (19.81–20.45)	26.59 (26.05–27.15)	38,987	7,236	18.56 (18.18–18.95)	25.03 (24.35–25.75)	20,893	4,818	23.06 (22.49–23.64)	29.11 (28.22–30.03)	12.32	**< 0.001**	< 0.1
Nayarit	101,399	16,689	16.46 (16.23–16.69)	22.55 (22.17–22.94)	67,250	10,227	15.21 (14.94–15.48)	20.92 (20.45–21.4)	34,149	6,462	18.92 (18.51–19.34)	25.66 (24.99–26.35)	0.18	0.67	< 0.1
Nuevo Leon	534,527	111,494	20.86 (20.75–20.97)	26.89 (26.72–27.07)	339,820	66,414	19.54 (19.41–19.68)	25.52 (25.29–25.75)	194,707	45,080	23.15 (22.97–23.34)	29.18 (28.89–29.48)	12.02	**< 0.001**	< 0.1
Oaxaca	67,160	14,580	21.71 (21.4–22.02)	27.38 (26.88–27.89)	43,080	8,710	20.22 (19.84–20.6)	26.32 (25.67–26.99)	24,080	5,870	24.38 (23.84–24.92)	29.22 (28.43–30.04)	0.09	0.77	< 0.1
Puebla	354,046	117,799	33.27 (33.12–33.43)	38.15 (37.91–38.4)	219,737	68,686	31.26 (31.06–31.45)	36.34 (36.03–36.67)	134,309	49,113	36.57 (36.31–36.83)	40.91 (40.52–41.3)	1.70	0.19	< 0.1
Queretaro	230,140	71,270	30.97 (30.78–31.16)	35.75 (35.45–36.05)	140,355	41,169	29.33 (29.09–29.57)	34.23 (33.84–34.62)	89,785	30,101	33.53 (33.22–33.84)	37.96 (37.49–38.43)	11.74	**< 0.001**	< 0.1
Quintana Roo	90,541	19,362	21.38 (21.12–21.65)	26.12 (25.69–26.57)	57,723	11,237	19.47 (19.14–19.79)	24.18 (23.63–24.75)	32,818	8,125	24.76 (24.29–25.23)	29.39 (28.67–30.12)	10.27	**0.001**	< 0.1
San Luis Potosi	138,236	37,687	27.26 (27.03–27.5)	32.5 (32.14–32.85)	88,682	22,860	25.78 (25.49–26.07)	31.14 (30.68–31.6)	49,554	14,827	29.92 (29.52–30.33)	34.83 (34.25–35.42)	4.88	**0.03**	< 0.1
Sinaloa	470,240	73,340	15.6 (15.49–15.7)	20.97 (20.8–21.14)	306,701	44,981	14.67 (14.54–14.79)	19.62 (19.41–19.84)	163,539	28,359	17.34 (17.16–17.53)	23.62 (23.33–23.92)	40.45	**< 0.001**	< 0.1
Sonora	356,610	75,211	21.09 (20.96–21.22)	27.06 (26.85–27.28)	228,870	45,469	19.87 (19.7–20.03)	25.63 (25.36–25.9)	127,740	29,742	23.28 (23.05–23.52)	29.63 (29.27–29.99)	25.90	**< 0.001**	< 0.1
Tabasco	102,333	15,131	14.79 (14.57–15.01)	20.13 (19.75–20.51)	67,646	8,836	13.06 (12.81–13.32)	18.42 (17.94–18.9)	34,687	6,295	18.15 (17.74–18.56)	23.17 (22.54–23.82)	61.14	**< 0.001**	< 0.1
Tamaulipas	214,611	36,660	17.08 (16.92–17.24)	25.44 (25.14–25.75)	142,205	21,909	15.41 (15.22–15.6)	23.59 (23.22–23.97)	72,406	14,751	20.37 (20.08–20.67)	28.79 (28.28–29.3)	90.12	**< 0.001**	< 0.1
Tlaxcala	29,111	11,477	39.42 (38.86–39.99)	45.16 (44.26–46.07)	18,169	6,789	37.37 (36.66–38.07)	43.53 (42.37–44.74)	10,942	4,688	42.84 (41.91–43.78)	47.76 (46.33–49.24)	6.80	**0.009**	< 0.1
Veracruz	398,163	76,929	19.32 (19.2–19.44)	25.24 (25.04–25.43)	255,147	44,740	17.53 (17.39–17.68)	23.28 (23.03–23.53)	143,016	32,189	22.51 (22.29–22.72)	28.54 (28.21–28.88)	137.38	**< 0.001**	< 0.1
Yucatan	112,344	21,842	19.44 (19.21–19.67)	25.3 (24.91–25.69)	72,688	12,590	17.32 (17.05–17.6)	23.07 (22.58–23.57)	39,656	9,252	23.33 (22.92–23.75)	28.88 (28.24–29.54)	103.66	**< 0.001**	< 0.1
Zacatecas	60,509	19,208	31.74 (31.37–32.12)	36.78 (36.22–37.35)	38,004	11,678	30.73 (30.26–31.19)	35.82 (35.1–36.55)	22,505	7,530	33.46 (32.84–34.08)	38.24 (37.34–39.16)	17.77	**< 0.001**	< 0.1

CI, confidence interval; Cyl, cylinder power; D, diopter. The Chi-square test of independence was used to test the association between sex and the prevalence of refractive astigmatism. Effect size was estimated using Cramér's *V* coefficient (φ_c). A *p*-value < 0.05 was considered statistically significant; statistically significant values are shown in bold.

[a]Age-adjusted prevalence to the World Health Organization (WHO) world standard population.

[b]Cramér's *V* coefficient (φ_c)

Table S5. Crude and age-adjusted state-level prevalence estimates of against-the-rule (ATR) astigmatism among Mexican outpatients

State	Total individuals (*n*)	Against-the-rule (ATR) astigmatism													
		General			Female				Male						
		n	Crude % (95% CI)	Age-adjusted[a] % (95% CI)	Total	*n*	Crude % (95% CI)	Age-adjusted[a] % (95% CI)	Total	*n*	Crude % (95% CI)	Age-adjusted[a] % (95% CI)	χ^2	*p*-value	φ_c[b]
Aguascalientes	127,088	11,976	9.42 (9.26–9.59)	7.23 (7.09–7.37)	80,191	6,781	8.46 (8.26–8.65)	6.44 (6.27–6.61)	46,897	5,195	11.08 (10.79–11.37)	8.61 (8.37–8.87)	68.98	**< 0.001**	< 0.1
Baja California	573,401	61,899	10.8 (10.71–10.88)	8.52 (8.45–8.59)	357,516	35,419	9.91 (9.81–10.01)	7.87 (7.79–7.96)	215,885	26,480	12.27 (12.13–12.4)	9.6 (9.48–9.72)	65.37	**< 0.001**	< 0.1
Baja California Sur	34,902	4,404	12.62 (12.27–12.97)	9.34 (9.04–9.66)	22,662	2,510	11.08 (10.67–11.49)	8.38 (8.01–8.78)	12,240	1,894	15.47 (14.84–16.13)	11.18 (10.64–11.77)	24.34	**< 0.001**	< 0.1
Campeche	29,267	3,972	13.57 (13.18–13.97)	9.87 (9.54–10.22)	18,048	2,343	12.98 (12.49–13.48)	9.46 (9.03–9.93)	11,219	1,629	14.52 (13.87–15.19)	10.64 (10.09–11.22)	9.56	**0.002**	< 0.1
Chiapas	197,530	17,411	8.81 (8.69–8.94)	6.45 (6.35–6.56)	123,075	9,866	8.02 (7.87–8.17)	5.91 (5.78–6.04)	74,455	7,545	10.13 (9.92–10.35)	7.36 (7.18–7.54)	0.70	0.40	< 0.1
Chihuahua	209,538	22,166	10.58 (10.45–10.71)	7.54 (7.43–7.65)	134,995	13,138	9.73 (9.57–9.89)	6.77 (6.64–6.9)	74,543	9,028	12.11 (11.88–12.35)	8.96 (8.76–9.17)	51.50	**< 0.001**	< 0.1
Mexico City	961,200	97,464	10.14 (10.08–10.2)	6.65 (6.61–6.7)	611,102	58,645	9.6 (9.52–9.67)	6.11 (6.06–6.17)	350,098	38,819	11.09 (10.98–11.19)	7.68 (7.59–7.76)	20.04	**< 0.001**	< 0.1
Coahuila	180,639	15,801	8.75 (8.62–8.88)	6.66 (6.55–6.78)	116,404	9,245	7.94 (7.79–8.1)	6.04 (5.91–6.18)	64,235	6,556	10.21 (9.97–10.44)	7.79 (7.59–8)	60.50	**< 0.001**	< 0.1
Colima	69,345	8,461	12.2 (11.96–12.45)	7.85 (7.66–8.04)	43,308	4,885	11.28 (10.98–11.58)	7.24 (7.01–7.48)	26,037	3,576	13.73 (13.32–14.16)	8.92 (8.6–9.27)	0.16	0.69	< 0.1
Durango	144,636	11,413	7.89 (7.75–8.03)	5.88 (5.76–5.99)	94,491	6,623	7.01 (6.85–7.17)	5.25 (5.11–5.39)	50,145	4,790	9.55 (9.3–9.81)	7.07 (6.86–7.29)	58.20	**< 0.001**	< 0.1
State of Mexico	1,358,039	119,308	8.79 (8.74–8.83)	6.72 (6.68–6.76)	843,776	69,833	8.28 (8.22–8.34)	6.2 (6.15–6.25)	514,263	49,475	9.62 (9.54–9.7)	7.65 (7.58–7.72)	23.03	**< 0.001**	< 0.1
Guanajuato	435,611	39,031	8.96 (8.88–9.05)	6.81 (6.74–6.88)	271,978	21,973	8.08 (7.98–8.18)	6.04 (5.95–6.13)	163,633	17,058	10.42 (10.28–10.57)	8.14 (8.01–8.27)	93.90	**< 0.001**	< 0.1
Guerrero	85,456	12,869	15.06 (14.82–15.3)	9.8 (9.6–10.01)	56,684	8,214	14.49 (14.2–14.78)	9.41 (9.17–9.67)	28,772	4,655	16.18 (15.76–16.61)	10.64 (10.29–11.01)	55.02	**< 0.001**	< 0.1
Hidalgo	62,559	4,464	7.14 (6.94–7.34)	5.1 (4.94–5.27)	39,010	2,536	6.5 (6.26–6.75)	4.6 (4.41–4.81)	23,549	1,928	8.19 (7.84–8.54)	5.98 (5.7–6.27)	5.15	**0.02**	< 0.1
Jalisco	647,682	65,393	10.1 (10.02–10.17)	7.53 (7.47–7.6)	408,946	37,346	9.13 (9.04–9.22)	6.73 (6.66–6.8)	238,736	28,047	11.75 (11.62–11.88)	8.95 (8.84–9.06)	228.35	**< 0.001**	< 0.1
Michoacan	185,448	16,750	9.03 (8.9–9.16)	6.27 (6.16–6.37)	119,112	9,832	8.25 (8.1–8.41)	5.73 (5.61–5.86)	66,336	6,918	10.43 (10.2–10.66)	7.25 (7.06–7.44)	15.10	**< 0.001**	< 0.1
Morelos	59,880	6,679	11.15 (10.9–11.41)	7.06 (6.86–7.26)	38,987	4,142	10.62 (10.32–10.93)	6.54 (6.31–6.79)	20,893	2,537	12.14 (11.7–12.59)	8.04 (7.69–8.41)	4.14	**0.04**	< 0.1
Nayarit	101,399	9,940	9.8 (9.62–9.99)	6.57 (6.43–6.72)	67,250	6,042	8.98 (8.77–9.2)	6.08 (5.91–6.26)	34,149	3,898	11.41 (11.08–11.76)	7.6 (7.33–7.88)	1.00	0.32	< 0.1
Nuevo Leon	534,527	57,560	10.77 (10.69–10.85)	7.7 (7.63–7.77)	339,820	34,397	10.12 (10.02–10.22)	7.15 (7.06–7.23)	194,707	23,163	11.9 (11.75–12.04)	8.69 (8.57–8.81)	0.53	0.47	< 0.1
Oaxaca	67,160	4,946	7.36 (7.17–7.56)	5.15 (5–5.32)	43,080	2,916	6.77 (6.53–7.01)	4.71 (4.52–4.92)	24,080	2,030	8.43 (8.08–8.79)	5.97 (5.69–6.26)	1.39	0.24	< 0.1
Puebla	354,046	31,979	9.03 (8.94–9.13)	6.77 (6.69–6.85)	219,737	18,490	8.41 (8.3–8.53)	6.24 (6.14–6.33)	134,309	13,489	10.04 (9.88–10.21)	7.7 (7.56–7.84)	5.63	**0.02**	< 0.1
Queretaro	230,140	24,529	10.66 (10.53–10.79)	8.01 (7.91–8.12)	140,355	13,704	9.76 (9.61–9.92)	7.25 (7.12–7.38)	89,785	10,825	12.06 (11.84–12.27)	9.26 (9.08–9.44)	31.43	**< 0.001**	< 0.1
Quintana Roo	90,541	10,495	11.59 (11.38–11.8)	8.9 (8.72–9.09)	57,723	6,266	10.86 (10.6–11.11)	8.32 (8.1–8.56)	32,818	4,229	12.89 (12.53–13.25)	9.93 (9.62–10.27)	5.98	**0.01**	< 0.1
San Luis Potosi	138,236	10,061	7.28 (7.14–7.42)	5.6 (5.48–5.72)	88,682	5,837	6.58 (6.42–6.75)	5.02 (4.89–5.16)	49,554	4,224	8.52 (8.28–8.77)	6.67 (6.46–6.89)	29.04	**< 0.001**	< 0.1
Sinaloa	470,240	60,850	12.94 (12.84–13.04)	8.51 (8.44–8.59)	306,701	35,920	11.71 (11.6–11.83)	7.93 (7.84–8.02)	163,539	24,930	15.24 (15.07–15.42)	9.64 (9.5–9.77)	99.72	**< 0.001**	< 0.1
Sonora	356,610	33,723	9.46 (9.36–9.55)	6.86 (6.79–6.94)	228,870	19,521	8.53 (8.42–8.64)	6.25 (6.16–6.35)	127,740	14,202	11.12 (10.95–11.29)	7.98 (7.84–8.13)	81.56	**< 0.001**	< 0.1
Tabasco	102,333	13,386	13.08 (12.87–13.29)	9.4 (9.22–9.58)	67,646	8,424	12.45 (12.21–12.7)	8.89 (8.68–9.11)	34,687	4,962	14.31 (13.94–14.68)	10.44 (10.13–10.76)	54.47	**< 0.001**	< 0.1
Tamaulipas	214,611	24,853	11.58 (11.45–11.72)	7.62 (7.52–7.73)	142,205	15,740	11.07 (10.91–11.23)	7.21 (7.08–7.35)	72,406	9,113	12.59 (12.35–12.83)	8.48 (8.29–8.68)	66.12	**< 0.001**	< 0.1
Tlaxcala	29,111	3,027	10.4 (10.05–10.75)	7.82 (7.53–8.13)	18,169	1,811	9.97 (9.54–10.41)	7.46 (7.09–7.86)	10,942	1,216	11.11 (10.53–11.72)	8.47 (7.98–9)	0.00	0.96	< 0.1
Veracruz	398,163	53,879	13.53 (13.43–13.64)	8.87 (8.79–8.95)	255,147	33,006	12.94 (12.81–13.07)	8.4 (8.3–8.5)	143,016	20,873	14.59 (14.41–14.78)	9.75 (9.6–9.89)	107.77	**< 0.001**	< 0.1
Yucatan	112,344	16,425	14.62 (14.41–14.83)	10.1 (9.93–10.28)	72,688	10,299	14.17 (13.92–14.42)	9.56 (9.35–9.77)	39,656	6,126	15.45 (15.09–15.81)	11.16 (10.86–11.47)	87.35	**< 0.001**	< 0.1
Zacatecas	60,509	5,851	9.67 (9.44–9.91)	7.18 (6.99–7.38)	38,004	3,267	8.6 (8.32–8.88)	6.34 (6.11–6.59)	22,505	2,584	11.48 (11.07–11.91)	8.68 (8.34–9.04)	53.51	**< 0.001**	< 0.1

CI, confidence interval; Cyl, cylinder power; D, diopter. The Chi-square test of independence was used to test the association between sex and the prevalence of refractive astigmatism. Effect size was estimated using Cramér's *V* coefficient (φ_c). A *p*-value < 0.05 was considered statistically significant; statistically significant values are shown in bold.

[a]Age-adjusted prevalence to the World Health Organization (WHO) world standard population.

[b]Cramér's *V* coefficient (φ_c)

Table S6. Crude and age-adjusted state-level prevalence estimates of oblique (OBL) astigmatism among Mexican outpatients

State	Total individuals (***n***)	Oblique (OBL) astigmatism														
		General			Female				Male							
		n	Crude % (95% CI)	Age-adjusted[a] % (95% CI)	Total	*n*	Crude % (95% CI)	Age-adjusted[a] % (95% CI)	**Total**	*n*	Crude % (95% CI)	Age-adjusted[a] % (95% CI)	χ^2	*p*-value	φ_c[b]
Aguascalientes	127,088	3,953	3.11 (3.02–3.21)	2.78 (2.69–2.88)	80,191	2,453	3.06 (2.94–3.18)	2.7 (2.59–2.83)	46,897	1,500	3.2 (3.04–3.36)	2.92 (2.77–3.09)	8.60	**0.003**	< 0.1
Baja California	573,401	12,560	2.19 (2.15–2.23)	1.98 (1.94–2.02)	357,516	7,674	2.15 (2.1–2.19)	1.92 (1.87–1.96)	215,885	4,886	2.26 (2.2–2.33)	2.09 (2.02–2.15)	35.44	**< 0.001**	< 0.1
Baja California Sur	34,902	782	2.24 (2.09–2.4)	1.96 (1.8–2.13)	22,662	498	2.2 (2.01–2.4)	1.87 (1.69–2.1)	12,240	284	2.32 (2.06–2.6)	2.12 (1.86–2.44)	4.87	**0.03**	< 0.1
Campeche	29,267	518	1.77 (1.62–1.93)	1.44 (1.31–1.6)	18,048	292	1.65 (1.47–1.85)	1.41 (1.24–1.6)	11,219	226	2.01 (1.76–2.29)	1.72 (1.48–2)	0.11	0.75	< 0.1
Chiapas	197,530	2,949	1.49 (1.44–1.55)	1.34 (1.28–1.39)	123,075	1,699	1.38 (1.32–1.45)	1.22 (1.16–1.3)	74,455	1,250	1.68 (1.59–1.77)	1.53 (1.44–1.63)	0.61	0.43	< 0.1
Chihuahua	209,538	6,129	2.93 (2.85–3)	2.56 (2.49–2.63)	134,995	3,958	2.93 (2.84–3.02)	2.47 (2.38–2.56)	74,543	2,171	2.91 (2.79–3.04)	2.68 (2.56–2.81)	30.86	**< 0.001**	< 0.1
Mexico City	961,200	32,652	3.4 (3.36–3.43)	2.78 (2.75–2.82)	611,102	20,891	3.42 (3.37–3.46)	2.69 (2.65–2.74)	350,098	11,761	3.36 (3.3–3.42)	2.94 (2.88–3)	149.72	**< 0.001**	< 0.1
Coahuila	180,639	4,563	2.53 (2.45–2.6)	2.31 (2.23–2.38)	116,404	2,919	2.51 (2.42–2.6)	2.28 (2.18–2.37)	64,235	1,644	2.56 (2.44–2.68)	2.37 (2.25–2.5)	16.35	**< 0.001**	< 0.1
Colima	69,345	1,264	1.82 (1.72–1.93)	1.5 (1.41–1.61)	43,308	766	1.77 (1.65–1.9)	1.42 (1.3–1.55)	26,037	498	1.91 (1.75–2.09)	1.64 (1.48–1.83)	4.05	**0.04**	< 0.1
Durango	144,636	3,369	2.33 (2.25–2.41)	2.12 (2.04–2.2)	94,491	2,114	2.24 (2.14–2.33)	1.99 (1.9–2.09)	50,145	1,255	2.5 (2.37–2.64)	2.35 (2.21–2.5)	4.27	**0.04**	< 0.1
State of Mexico	1,358,039	40,742	3 (2.97–3.03)	2.64 (2.62–2.67)	843,776	25,387	3.01 (2.97–3.05)	2.57 (2.54–2.61)	514,263	15,355	2.99 (2.94–3.03)	2.76 (2.71–2.8)	179.66	**< 0.001**	< 0.1
Guanajuato	435,611	12,064	2.77 (2.72–2.82)	2.44 (2.39–2.49)	271,978	7,406	2.72 (2.66–2.78)	2.33 (2.28–2.39)	163,633	4,658	2.85 (2.77–2.93)	2.6 (2.52–2.68)	47.06	**< 0.001**	< 0.1
Guerrero	85,456	1,568	1.83 (1.75–1.93)	1.52 (1.43–1.62)	56,684	965	1.7 (1.6–1.81)	1.36 (1.25–1.48)	28,772	603	2.1 (1.93–2.27)	1.85 (1.68–2.05)	0.00	0.97	< 0.1
Hidalgo	62,559	1,786	2.85 (2.73–2.99)	2.44 (2.32–2.57)	39,010	1,080	2.77 (2.61–2.94)	2.33 (2.17–2.49)	23,549	706	3 (2.78–3.22)	2.63 (2.43–2.86)	3.62	0.06	< 0.1
Jalisco	647,682	16,955	2.62 (2.58–2.66)	2.27 (2.23–2.3)	408,946	10,368	2.54 (2.49–2.58)	2.14 (2.09–2.19)	238,736	6,587	2.76 (2.69–2.83)	2.47 (2.41–2.54)	18.82	**< 0.001**	< 0.1
Michoacan	185,448	4,128	2.23 (2.16–2.29)	2.01 (1.94–2.08)	119,112	2,577	2.16 (2.08–2.25)	1.91 (1.83–2)	66,336	1,551	2.34 (2.22–2.46)	2.18 (2.06–2.31)	11.35	**< 0.001**	< 0.1
Morelos	59,880	1,509	2.52 (2.4–2.65)	2.01 (1.89–2.14)	38,987	974	2.5 (2.35–2.66)	1.89 (1.75–2.05)	20,893	535	2.56 (2.35–2.78)	2.22 (2.01–2.45)	8.52	**0.004**	< 0.1
Nayarit	101,399	1,530	1.51 (1.43–1.59)	1.42 (1.34–1.51)	67,250	958	1.42 (1.34–1.52)	1.31 (1.21–1.42)	34,149	572	1.68 (1.54–1.82)	1.65 (1.5–1.82)	1.41	0.24	< 0.1
Nuevo Leon	534,527	10,774	2.02 (1.98–2.05)	1.79 (1.75–1.83)	339,820	6,872	2.02 (1.98–2.07)	1.74 (1.69–1.79)	194,707	3,902	2 (1.94–2.07)	1.87 (1.81–1.94)	72.64	**< 0.001**	< 0.1
Oaxaca	67,160	1,180	1.76 (1.66–1.86)	1.52 (1.43–1.63)	43,080	730	1.69 (1.57–1.82)	1.42 (1.31–1.55)	24,080	450	1.87 (1.7–2.05)	1.69 (1.53–1.88)	2.50	0.11	< 0.1
Puebla	354,046	10,035	2.83 (2.78–2.89)	2.43 (2.38–2.48)	219,737	6,161	2.8 (2.74–2.87)	2.36 (2.29–2.42)	134,309	3,874	2.88 (2.8–2.98)	2.54 (2.46–2.63)	39.44	**< 0.001**	< 0.1
Queretaro	230,140	7,045	3.06 (2.99–3.13)	2.6 (2.54–2.67)	140,355	4,173	2.97 (2.88–3.06)	2.5 (2.42–2.58)	89,785	2,872	3.2 (3.08–3.32)	2.77 (2.67–2.88)	10.25	**0.001**	< 0.1
Quintana Roo	90,541	1,835	2.03 (1.94–2.12)	1.73 (1.65–1.83)	57,723	1,114	1.93 (1.82–2.05)	1.62 (1.51–1.74)	32,818	721	2.2 (2.04–2.36)	1.93 (1.78–2.1)	3.10	0.08	< 0.1
San Luis Potosi	138,236	3,435	2.48 (2.4–2.57)	2.28 (2.2–2.36)	88,682	2,203	2.48 (2.38–2.59)	2.22 (2.12–2.33)	49,554	1,232	2.49 (2.35–2.63)	2.37 (2.23–2.52)	21.79	**< 0.001**	< 0.1
Sinaloa	470,240	8,926	1.9 (1.86–1.94)	1.66 (1.62–1.7)	306,701	5,728	1.87 (1.82–1.92)	1.61 (1.57–1.66)	163,539	3,198	1.96 (1.89–2.02)	1.76 (1.69–1.84)	52.84	**< 0.001**	< 0.1
Sonora	356,610	7,055	1.98 (1.93–2.02)	1.79 (1.74–1.84)	228,870	4,506	1.97 (1.91–2.03)	1.76 (1.7–1.82)	127,740	2,549	2 (1.92–2.07)	1.87 (1.79–1.95)	48.89	**< 0.001**	< 0.1
Tabasco	102,333	1,906	1.86 (1.78–1.95)	1.61 (1.53–1.7)	67,646	1,176	1.74 (1.64–1.84)	1.5 (1.4–1.62)	34,687	730	2.1 (1.96–2.26)	1.82 (1.67–1.98)	1.03	0.31	< 0.1
Tamaulipas	214,611	3,556	1.66 (1.6–1.71)	1.44 (1.38–1.5)	142,205	2,276	1.6 (1.54–1.67)	1.37 (1.3–1.45)	72,406	1,280	1.77 (1.67–1.87)	1.56 (1.46–1.66)	11.11	**< 0.001**	< 0.1
Tlaxcala	29,111	1,458	5.01 (4.76–5.27)	4.44 (4.19–4.71)	18,169	943	5.19 (4.87–5.52)	4.45 (4.14–4.8)	10,942	515	4.71 (4.32–5.12)	4.34 (3.94–4.78)	15.97	**< 0.001**	< 0.1
Veracruz	398,163	8,014	2.01 (1.97–2.06)	1.65 (1.61–1.69)	255,147	4,912	1.93 (1.87–1.98)	1.54 (1.49–1.59)	143,016	3,102	2.17 (2.09–2.25)	1.84 (1.77–1.92)	10.82	**0.001**	< 0.1
Yucatan	112,344	2,511	2.24 (2.15–2.32)	1.85 (1.77–1.94)	72,688	1,554	2.14 (2.03–2.25)	1.71 (1.61–1.83)	39,656	957	2.41 (2.26–2.57)	2.1 (1.95–2.26)	4.22	**0.04**	< 0.1
Zacatecas	60,509	2,183	3.61 (3.46–3.76)	3.23 (3.08–3.38)	38,004	1,397	3.68 (3.49–3.87)	3.19 (3.01–3.38)	22,505	786	3.49 (3.26–3.74)	3.27 (3.03–3.52)	15.87	**< 0.001**	< 0.1

CI, confidence interval; Cyl, cylinder power; D, diopter. The Chi-square test of independence was used to test the association between sex and the prevalence of refractive astigmatism. Effect size was estimated using Cramér's *V* coefficient (φ_c). A *p*-value < 0.05 was considered statistically significant; statistically significant values are shown in bold.

[a]Age-adjusted prevalence to the World Health Organization (WHO) world standard population.

[b]Cramér's *V* coefficient (φ_c)
